# Integrative multi-omics analysis and machine learning reveal the unique role of ASCC3 in combination with various immune-related genes in rectal adenocarcinoma

**DOI:** 10.3389/fgene.2025.1614946

**Published:** 2025-08-13

**Authors:** Chuang Miao, Fei Qian

**Affiliations:** Department of General Surgery, Affiliated Hospital of Nantong University, Medical School of Nantong University, Nantong, China

**Keywords:** READ, ASCC3, machine learning, prognosis, tumor immunity, DNA damage repair, genome stability

## Abstract

**Background:**

The Activating Signal Cointegrator 1 (ASC-1) complex is a tetrameric complex composed of Thyroid Hormone Receptor Interactor 4 (TRIP4), Activating Signal Cointegrator 1 (ASCC1), Activating Signal Cointegrator 2 (ASCC2), and Activating Signal Cointegrator 3 (ASCC3). As the core subunit of the ASC-1 complex, ASCC3 is involved in DNA damage repair. However, the exact role of ASCC3 in digestive system cancers, particularly in rectal adenocarcinoma (READ), remains unclear.

**Methods:**

We utilized data from The Cancer Genome Atlas (TCGA) and GEO to investigate the prognostic value of the four subunits, with a focus on ASCC3, in pan-cancers including rectal adenocarcinoma. Additionally, we explored the regulatory mechanisms of ASCC3 and its association with tumor immunity in rectal adenocarcinoma using TIMER, IMMPORT, DAVID databases, and CIBERSORT analysis. Prognostic models were constructed using ssGSEA analysis and various machine learning algorithms. We explored the signaling pathways regulated by ASCC3 using the CancerSEA and TCPA databases. Furthermore, mutations of ASCC3 in pan-cancer were investigated using the PFAM and CbioPortal databases. Finally, we performed interaction network analysis of ASCC3 using STRING, ComPPI, and BioGRID databases.

**Results:**

ASCC3 is a specific protective factor for rectal adenocarcinoma across various cancer types, particularly in digestive system cancers, and can synergize with several immune-related genes, including JAK1, NFKB1, SEMA5A, NR2C2, CNTF, and CREB1, to influence patient prognosis. Furthermore, ASCC3 may regulate tumor immunity by affecting T cell function.

**Conclusion:**

ASCC3 can serve as an independent prognostic factor for READ and can synergize with various immune-related genes to influence patient prognosis.

## 1 Introduction

According to the latest cancer statistics, the incidence and mortality rates of digestive system cancers are the highest globally, with colorectal cancer, gastric cancer, esophageal cancer, liver cancer, and others ranking among the top ten. The incidence of colorectal cancer is continuously increasing, with a trend towards younger age groups (Siegel et al., 2025). Due to the subtle onset of symptoms in colorectal cancer, most cases are diagnosed at advanced stages, leading to poor treatment outcomes and a significant reduction in median survival time (Tonini and Zanni, 2024; Zygulska and Pierzchalski, 2022). Recent studies on molecular mechanisms have revealed the critical role of aberrant activation of key signaling pathways such as the Wnt pathway in tumorigenesis (Zhou et al., 2022). Molecular features like mismatch repair deficiency (dMMR) or microsatellite instability-high (MSI-H) have provided new directions for precision therapy (Zhao et al., 2019). Additionally, the association between gut microbiota dysbiosis and immune escape has opened new perspectives for studying the pathogenesis (Wang and Fang, 2023). However, the effectiveness of these therapies remains limited for patients in advanced stages, especially for those with low-lying rectal cancer, who often lose their anus as a result, significantly affecting their postoperative quality of life (Mei et al., 2022; Ye et al., 2022). Therefore, there is an urgent need to identify specific molecular markers associated with rectal cancer to enhance early diagnostic capabilities and assist in the effective treatment of rectal cancer.

The ASC-1 complex is a tetrameric complex composed of TRIP4, ASCC1, ASCC2, and ASCC3, initially identified as a transcriptional coactivator for steroid receptors. It has now been found to promote the dissociation of the 80S ribosome and interact with scanning ribosomes to regulate translation initiation (Juszkiewicz et al., 2020; Kim et al., 1999; Kito et al., 2023). TRIP4 has been reported to promote gliomas and cervical cancer and may serve as a potential target for immunotherapy in colon cancer (Che et al., 2019; Li et al., 2021; Scanlan et al., 2002). Gene mutations in ASCC1 have been reported to be associated with prenatal fractures and the occurrence of barrett esophagus and esophageal adenocarcinoma (Meunier et al., 2021; Orloff et al., 2011). ASCC2 is associated with DNA alkylation damage (Brickner et al., 2017; Lombardi et al., 2022).

The ASCC3 subunit is a double-box Ski2-like RNA helicase involved in DNA damage repair. It provides single-stranded DNA for the repair of alkylation damage by the α-ketoglutarate-dependent dioxygenase AlkBH3, and is integrated into the complex DNA repair pathway by other ASCC components (Jia J. et al., 2020). As the core subunit of the ASC-1 complex, ASCC3 has been previously reported to promote immune suppression and progression in non-small cell lung cancer (Ao et al., 2023). However, the exact role of ASCC3 in digestive system cancers, particularly in rectal adenocarcinoma, remains unclear. Therefore, this study employs multi-omics analysis and machine learning to thoroughly investigate the prognostic potential and mechanisms of ASCC3 in rectal cancer. We found that high expression of ASCC3 in rectal cancer patients is associated with better prognosis. ASCC3 can serve as an independent prognostic factor for rectal cancer patients, particularly in younger individuals. Moreover, high expression of ASCC3 in rectal cancer can protect patients through its unique DNA damage repair functions and by synergizing with various immune-related genes.

## 2 Materials and methods

### 2.1 Data acquisition

Expression profiles of TRIP4, ASCC1, ASCC2, and ASCC3, along with clinical and expression data for ASCC3, were obtained from the TCGA database (http://gdc.cancer.gov/). Some clinical data for ASCC3 used in diagnosis were sourced from the GTEx database (https://www.gtexportal.org/). Immunohistochemical images of TRIP4, ASCC1, ASCC2, and ASCC3 in rectal cancer and normal tissue samples were obtained from the HPA database (https://www.proteinatlas.org/). The GSE87211 rectal cancer sample dataset was obtained from the GEO database (https://www.ncbi.nlm.nih.gov/geo/). The EMTAB8107 dataset was sourced from the EMBL-EBI database (https://www.ebi.ac.uk/). Immune-related gene (IRG) data were obtained from the IMMPORT database (https://immport.org/).

### 2.2 Data analysis and processing

**FIGURE 1:**
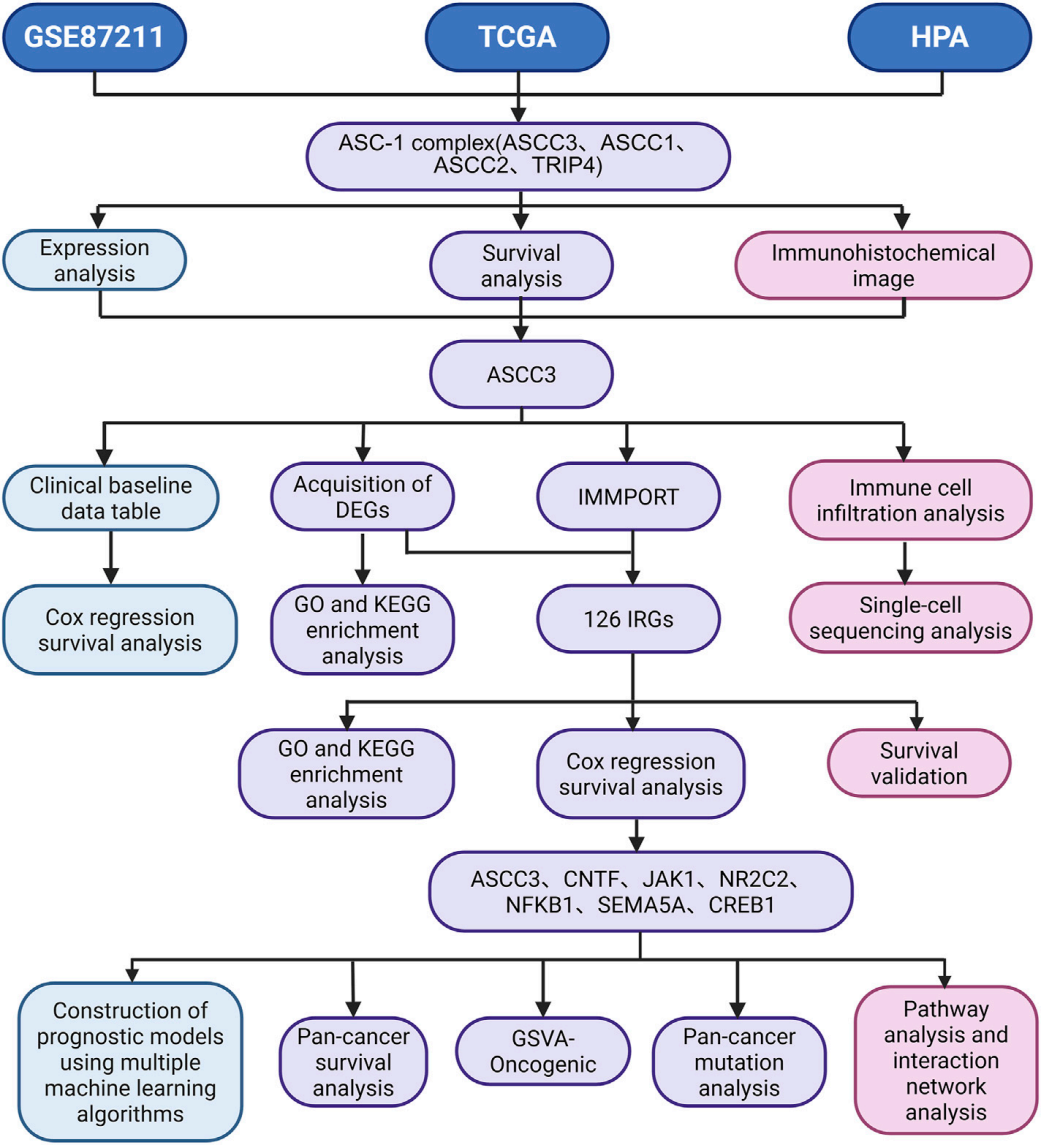
Study flowchart. TCGA, The Cancer Genome Atlas; GEO, Gene Expression Omnibus Series; HPA, Human Protein Atlas; ASC-1, The Activating Signal Cointegrator 1; TRIP4, Thyroid Hormone Receptor Interactor 4; ASCC1, Activating Signal Cointegrator 1; ASCC2, Activating Signal Cointegrator 2; ASCC3, Activating Signal Cointegrator 3; GO, Gene Ontology; KEGG, Kyoto Encyclopedia of Genes and Genomes; IMMPORT, The Immunology Database and Analysis Portal; DGEs, Differentially Expressed Genes; IRGs, Immune-related Genes; GSVA, Gene Set Variation Analysis.

BioRender (https://app.biorender.com/) was used to create the workflow diagram for this study (Figure 1). A pan-cancer comparative expression analysis of ASCC3 was conducted using the TIMER online database (https://cistrome.shinyapps.io/timer/). The analysis was performed using R (V4.2.1), with data processing standardized to log2 (value + 1), and data visualization carried out using the ggplot2 [3.4.4] package. The expression levels of rectal cancer RNA-seq data downloaded and organized from the TCGA database and the GSE87211 dataset were compared using the Wilcoxon rank sum test, with statistical significance defined as a p-value <0.05. Receiver operating characteristic (ROC) curve analysis of ASCC3-related data was conducted using the pROC [1.18.0] package. Time-dependent ROC curve analysis was performed using the timeROC [0.4] package. For the prognostic nomogram, proportional hazards assumption tests and Cox regression analysis were performed using the survival [3.3.1] package, and nomogram-related models were constructed and visualized using the rms [6.3–0] package. The prognostic endpoint used was Overall Survival (OS). The relationship between ASCC3 expression and clinical features was evaluated using the Kruskal–Wallis test, with a significance threshold set at a p-value <0.05. The DESeq2 [1.36.0] package was used to perform differential analysis on the original Counts matrix from the TCGA database. The criteria for differentially expressed genes were set as | log2 (FC) | > 1 and p-value <0.05. The Venn diagram for gene intersections was generated using the VennDiagram [1.7.3] package.

### 2.3 Survival and prognostic analysis

The analysis of the expression of TRIP4, ASCC1, ASCC2, and ASCC3 with patient survival and the survival analysis related to ASCC3 expression and multi-gene constructed ssGSEA scores were performed using the survival [3.3.1] package for proportional hazards assumption testing and survival regression fitting. Grouping was performed based on the median value, with statistical methods including Logrank test and Cox regression test, and visualization was conducted using the survminer [0.4.9] and ggplot2 [3.4.4] packages. For subgroup analysis of clinical data of patients with ASCC3 expression, as described above, the Logrank test was used, with statistical significance defined as a p-value <0.05. For the analysis of ASCC3 expression and patient prognosis with clinical multivariable analysis, the survival [3.3.1] package was used for proportional hazards assumption testing, followed by univariate and multivariate Cox regression analysis. In the univariate analysis, samples with a p-value <0.05 were selected for multivariate Cox regression analysis and OS was used as the prognostic type. Univariate Cox regression analysis was uniformly applied for the prognostic evaluation of immune-related genes associated with ASCC3 and their prognostic significance in digestive system cancers. The Chi-square (Chisq) test was used for group comparison of rectal cancer sample information, with grouping based on the median ASCC3 expression, and statistical significance was defined as a p-value <0.05. Spearman correlation analysis was also used to assess the association between ASCC3 expression and multiple immune-related genes.

### 2.4 Immune infiltration analysis and single-cell sequencing analysis

The correlation between ASCC3 and six common immune cells in rectal cancer was analyzed using the TIMER online database. The immune infiltration of ASCC3 in 22 immune cell types was assessed using the CIBERSORTx website (https://cibersortx.stanford.edu/), with statistical analysis performed using the Wilcoxon rank sum test and Spearman correlation analysis. We performed analysis of single-cell data using Uniform Manifold Approximation and Projection (UMAP) technique, presenting high-dimensional data in a two-dimensional heatmap for gene expression visualization and localization.

### 2.5 Construction of multi-gene ssGSEA pathway scores

ASCC3 and its associated immune genes, including JAK1, NFKB1, SEMA5A, NR2C2, CNTF and CREB1, were combined into an independent gene set. The gsva function from the R software GSVA package was then used to perform ssGSEA analysis to construct the enrichment scores for this independent gene set.

### 2.6 Machine learning and prognostic models

The train function from the caret package was used to train various machine learning models. The explain function from the DALEX package was used to interpret these models. The predict function was applied to evaluate model accuracy and plot ROC curves. The variable importance function from DALEX was used to calculate variable importance, and residual inverse cumulative distribution and box plots were generated. Lasso regression was performed using the glmnet package with the family parameter set to cox for Cox proportional hazards modeling. The maximum iterations were set to 1000, and ten-fold cross-validation was done with the cv.glmnet function. The coefficients and feature names for the optimal λ value were extracted from the model, along with the non-zero coefficients and corresponding features. The risk score was calculated by multiplying each gene’s expression level by its regression coefficient and summing the products. Kaplan-Meier survival analysis was performed using the survival package. The cutoff values for high-risk and low-risk groups were based on the median risk score, and a log-rank test was used to assess significance between the groups.

### 2.7 Signaling pathways analysis

Gene Ontology (GO) and Kyoto Encyclopedia of Genes and Genomes (KEGG) enrichment analyses of the target genes were performed online using the DAVID database (https://davidbioinformatics.nih.gov/). The functional states of 14 different cancer cell types were organized using the CancerSEA database (http://biocc.hrbmu.edu.cn/CancerSEA/). The z-score parameter from the R package GSVA was used to calculate the gene set scores for the cell cycle functional gene set, yielding the combined z-score, which was then standardized using the scale function. The correlation between the genes and the cell cycle gene set scores was then calculated. The easier package was used to compute pathway scores based on the PROGENyR software package (V1.10.0), and the Wilcoxon rank-sum test was applied to compare pathway activity scores between grouped samples. Protein expression data from reverse-phase protein arrays were downloaded from the TCPA database (https://www.tcpaportal.org/tcpa/). The activity scores for 10 cancer-related pathways were calculated, and the Spearman correlation and p-values between the target genes and pathway activity scores were determined using the cor.test function. The wilcox.test function was used to calculate the differences in pathway activity scores between the high-expression and low-expression gene groups.

### 2.8 Pan-cancer mutation and interaction network analysis

A lollipop plot was generated using the lollipopPlot function from the maftools package, with protein domains sourced from the PFAM database (http://pfam.xfam.org/). The mutation frequency differences of ASCC3 across pan-cancer were plotted using the Cbioportal database (https://www.cbioportal.org/). Proteins interacting with ASCC3 were identified using the STRING (https://cn.string-db.org/) and ComPPI databases (https://comppi.linkgroup.hu/). Interaction data with ASCC3 as the source protein were retrieved from the BioGRID database (https://thebiogrid.org/). A list of all proteins directly interacting with ASCC3 was obtained and used to construct a protein interaction network centered on ASCC3 using the igraph package.

## 3 Results

### 3.1 The expression of the four subunits of the ASC-1 complex varies in rectal adenocarcinoma

**FIGURE 2:**
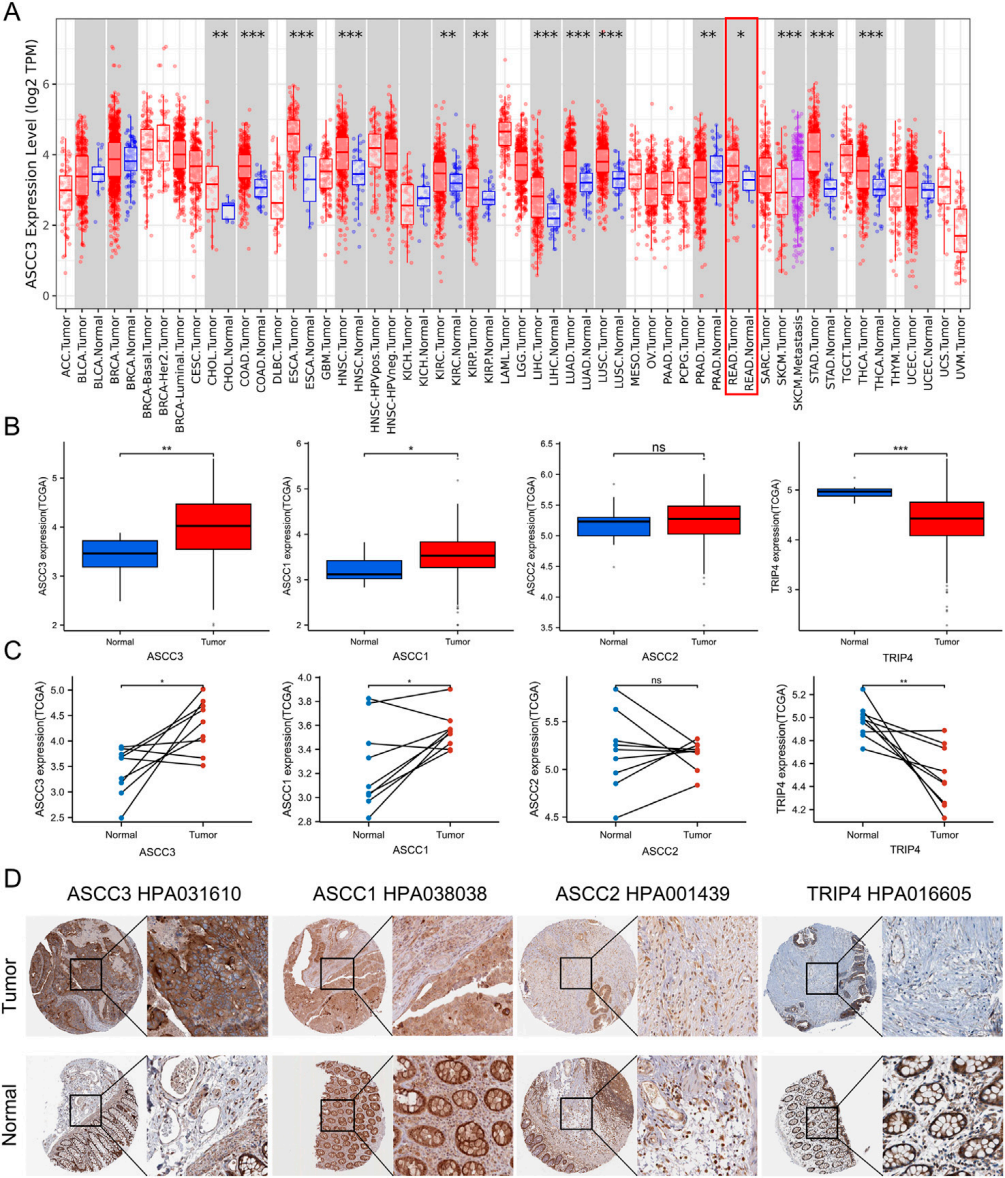
The expression of the four subunits of the ASC-1 complex in rectal adenocarcinoma (READ) and immunohistochemical images. **(A)** ASCC3 is highly expressed in various cancer types, including rectal adenocarcinoma. Statistical significance is indicated by asterisks (*p < 0.05; **p < 0.01; ***p < 0.001). **(B)** Expression of ASCC3, ASCC1, ASCC2, and TRIP4 in rectal tumor tissues compared to normal tissues in TCGA samples (*p < 0.05; **p < 0.01; ***p < 0.001; ns, not significant). **(C)** Paired comparison of the expression of ASCC3, ASCC1, ASCC2 and TRIP4 in rectal tumor tissues *versus* normal tissues in TCGA samples (*p < 0.05; **p < 0.01; ***p < 0.001; ns, not significant). **(D)** Immunohistochemical images of ASCC3, ASCC1, ASCC2, and TRIP4 in rectal tumor tissues and normal tissues from the HPA database.

Using TIMER database data, we analyzed the expression of ASCC3 gene in pan-cancer based on TCGA sample. As shown in Figure 2A, ASCC3 is highly expressed in various cancer types, including rectal adenocarcinoma, which is highlighted with a red box. Next, we analyzed the expression levels of ASCC3, ASCC1, ASCC2, and TRIP4 in TCGA rectal adenocarcinoma samples. The results showed that ASCC3 and ASCC1 is more highly expressed in tumor tissue, ASCC2 shows no difference between tumor and normal tissues, and TRIP4 is more significantly expressed in normal tissue (Figure 2B). Meanwhile, we performed a paired analysis of the expression levels of these four subunits in cancerous tissues and their corresponding normal tissues from the same patients, and the results were consistent with our previous findings (Figure 2C). To validate the above results, we analyzed the expression of the four subunits in the commonly used rectal cancer dataset GSE87211. The results slightly differed from those of TCGA, with ASCC3 and ASCC1 being more highly expressed in tumor tissue, while ASCC2 and TRIP4 were significantly expressed in normal tissue (Supplementary Figure S1). Finally, we collected immunohistochemical images of the four subunits in tumor and normal tissues using the same antibody from the HPA database (Figure 2D). The results showed that the comparative expression of ASCC3 and TRIP4 in tumor and normal tissues was consistent with the above findings, while the expression of ASCC1 and ASCC2 did not show clear differentiation.

### 3.2 ASCC3 demonstrates unique value in influencing the prognosis of rectal cancer patients

**FIGURE 3:**
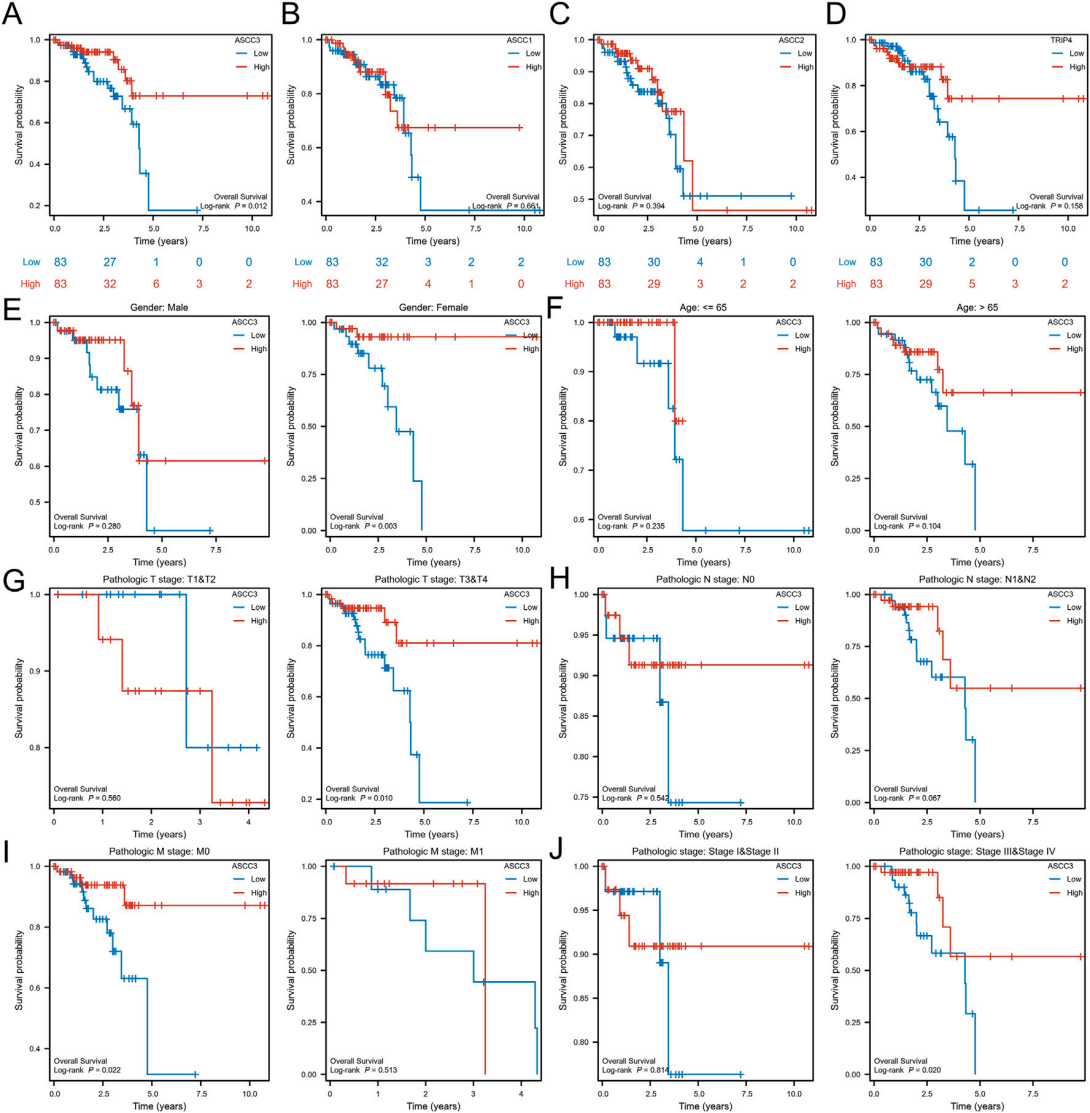
The association between the expression of the four subunits of the ASC-1 complex and overall survival (OS) in rectal cancer patients. **(A)** Patients with high ASCC3 expression in rectal cancer have better survival rates. **(B–D)** The expression of ASCC1, ASCC2 and TRIP4 in rectal cancer is not associated with patient survival. **(E–J)** Subgroup analysis of ASCC3 expression in rectal cancer in relation to patient gender, age, T stage, N stage, M stage, and pathological stage.

To explore the association between the four subunits of the ASC-1 complex and patient survival, we used a dual testing method comprising Log-rank and Cox regression tests. Based on the median ASCC3 expression level, we divided the samples into groups and assessed the correlation between the expression of ASCC3, ASCC1, ASCC2, and TRIP4 with OS in patients. As shown in Figures 3A–D and Supplementary Figure S2, the results indicate that among the 166 rectal cancer patient samples from the TCGA dataset, patients with high ASCC3 expression had significantly better survival rates (Log-rank test: p = 0.012; Cox regression test: HR = 0.36 (0.16–0.83), p = 0.016). The expression of the other three subunits showed no significant correlation with OS. Therefore, we infer that high ASCC3 expression may be a protective factor for rectal cancer patients. To further validate, we organized the baseline data of these 166 patients and divided them into groups based on the median ASCC3 expression level. As shown in Table 1, the Chi-square test revealed a significant association between ASCC3 expression and OS events (p = 0.033). To further explore the correlation between ASCC3 and these clinical data, we stratified the survival status of clinical information, including age, sex, T stage, N stage, M stage and pathological stage, into subgroups and conducted log-rank tests. The results showed that high ASCC3 expression was associated with better survival in subgroups of female patients, T3&T4 stage, M0 stage, and Stage III&IV patients (Figures 3E–J).

**TABLE 1:** Associations between ASCC3 expression and clinicopathological characteristics in 166 READ patients from the TCGA dataset.

Characteristics	Low expression of ASCC3	High expression of ASCC3	P Value
n	83	83	
Gender, n (%)			0.436
Male	43 (25.9%)	48 (28.9%)	
Female	40 (24.1%)	35 (21.1%)	
Age, n (%)			0.062
≤ 65	35 (21.1%)	47 (28.3%)	
>65	48 (28.9%)	36 (21.7%)	
Pathologic T stage, n (%)			0.575
T1&T2	17 (10.4%)	20 (12.2%)	
T3&T4	65 (39.6%)	62 (37.8%)	
Pathologic N stage, n (%)			0.870
N0	42 (25.9%)	42 (25.9%)	
N1&N2	40 (24.7%)	38 (23.5%)	
Pathologic M stage, n (%)			0.272
M0	65 (43.6%)	61 (40.9%)	
M1	9 (6%)	14 (9.4%)	
Pathologic stage, n (%)			0.873
Stage I&Stage II	41 (26.3%)	40 (25.6%)	
Stage III&Stage IV	37 (23.7%)	38 (24.4%)	
Histological type, n (%)			0.412
Adenocarcinoma	74 (46.2%)	73 (45.6%)	
Mucinous adenocarcinoma	5 (3.1%)	8 (5%)	
Perineural invasion, n (%)			0.202
Yes	2 (3.7%)	12 (22.2%)	
No	15 (27.8%)	25 (46.3%)	
Lymphatic invasion, n (%)			1.000
Yes	32 (21.6%)	32 (21.6%)	
No	42 (28.4%)	42 (28.4%)	
OS event, n (%)			0.033
Alive	65 (39.2%)	75 (45.2%)	
Dead	18 (10.8%)	8 (4.8%)	

### 3.3 ASCC3 can serve as an independent prognostic factor for rectal cancer patients

To further verify that ASCC3 expression is a favorable prognostic factor for rectal cancer patients, we performed Cox regression analysis on the clinical data of 166 rectal cancer cases from the above-mentioned group, including age, gender, T stage, N stage, M stage and pathological stage. As shown in Table 2, univariate regression revealed that age >65 years, N1 & N2 stage, M1 stage and Stage III & IV were unfavorable prognostic factors (HR > 1), whereas high ASCC3 expression was a protective factor for rectal cancer prognosis (HR < 1). Multivariate regression analysis showed that age >65 years could serve as an independent prognostic factor, while ASCC3 performed poorly, which is inconsistent with previous studies. Older patients (>65 years) are at higher risk for comorbidities, and the progression of existing comorbidities after cancer diagnosis significantly increases the probability of cancer-related death (Chen et al., 2022; Pliszka and Szablewski, 2024; Zhang et al., 2019). Moreover, due to ASCC3’s involvement in DNA repair functions, the genomic repair capacity and stability of older patients are greatly reduced (Kanungo, 2013; Sanchez-Roman et al., 2022), increasing their risk of cancer and mortality (Lord and Ashworth, 2012; Negrini et al., 2010). Thus, age could potentially absorb or mask some of the effect of ASCC3 expression on patient prognosis, or it may act as a mediating variable causing bias in the results. Given that our analysis of the association between age subgroups and survival did not show significant correlation and the Chi-square test for age was not significant, we excluded age as a baseline variable and performed univariate and multivariate Cox regression analysis again. As shown in Table 3, the results indicate that high ASCC3 expression is a favorable prognostic factor for patients, and ASCC3 can serve as an independent prognostic factor for rectal cancer patients.

**TABLE 2:** Univariate and multivariate Cox regression analysis of clinicopathologic variables in 166 READ patients.

Characteristics	Total(N)	Univariate analysis		Multivariate analysis	
		Hazard ratio (95% CI)	P Value	Hazard ratio (95% CI)	P Value
Gender	166	1.092 (0.503–2.370)	0.824		
Male	91				
Female	75				
Age	166	3.843 (1.535–9.622)	**0.004**	4.890 (1.620–14.758)	**0.005**
≤ 65	82				
>65	84				
T stage	164	1.408 (0.476–4.159)	0.536		
T1&T2	37				
T3&T4	127				
N stage	162	3.021 (1.244–7.336)	**0.015**	22993566.4098 (0.000 - Inf)	0.998
N0	84				
N1&N2	78				
M stage	149	3.412 (1.424–8.174)	**0.006**	2.695 (0.959–7.574)	0.060
M0	126				
M1	23				
Pathologic stage	156	3.380 (1.311–8.712)	**0.012**	0.000 (0.000 - Inf)	0.998
Stage Iand II	81				
Stage IIIand IV	75				
ASCC3	166	0.358 (0.155–0.829)	**0.016**	0.453 (0.182–1.129)	0.089
Low	83				
High	83				

Data with *p*-values less than 0.05 are highlighted in bold black.

**TABLE 3:** Univariate and multivariate Cox regression analysis of clinical pathological variables in 166 READ patients after excluding age.

Characteristics	Total(N)	Univariate analysis		Multivariate analysis	
		Hazard ratio (95% CI)	P Value	Hazard ratio (95% CI)	P Value
Gender	166	1.092 (0.503–2.370)	0.824		
Male	91				
Female	75				
T stage	164	1.408 (0.476–4.159)	0.536		
T1&T2	37				
T3&T4	127				
N stage	162	3.021 (1.244–7.336)	**0.015**	9581570.0816 (0.000 - Inf)	0.998
N0	84				
N1&N2	78				
M stage	149	3.412 (1.424–8.174)	**0.006**	2.142 (0.780–5.880)	0.139
M0	126				
M1	23				
Pathologic stage	156	3.380 (1.311–8.712)	**0.012**	0.000 (0.000 - Inf)	0.998
Stage Iand II	81				
Stage IIIand IV	75				
ASCC3	166	0.358 (0.155–0.829)	**0.016**	0.376 (0.152–0.927)	**0.034**
Low	83				
High	83				

Data with *p*-values less than 0.05 are highlighted in bold black.

### 3.4 The diagnostic value and clinical characteristics of ASCC3 in rectal cancer

**FIGURE 4:**
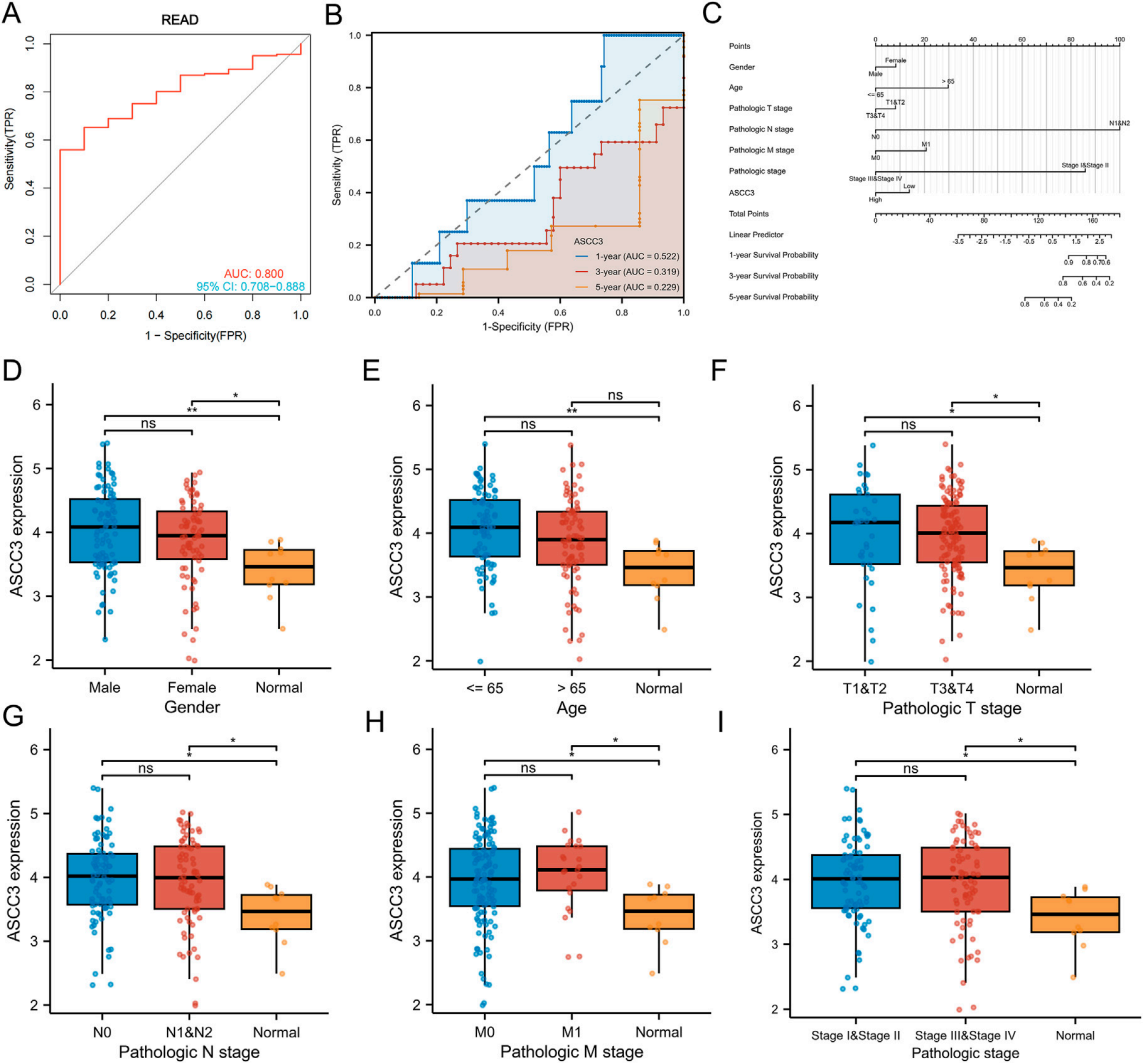
Diagnostic and prognostic efficacy of ASCC3 for rectal cancer and the expression of ASCC3 in different clinical baseline data types. **(A)** ROC curve showing the diagnostic accuracy of ASCC3 for rectal adenocarcinoma, with an AUC of 0.800 (95% CI: 0.708–0.888). **(B)** Time-dependent ROC diagnostic curve shows that the diagnostic ability of ASCC3 strengthens over time (When the value of a variable (protective factor) is opposite to the trend of event occurrence, a smaller area under the curve (AUC) value, closer to 0, indicates higher prognostic diagnostic value). **(C)** Nomogram for predicting 1-year, 3-year, and 5-year OS based on clinical factors such as age, sex, T stage, N stage, M stage, grade and ASCC3 expression levels. Each clinical factor is assigned a score, with the total score corresponding to the survival probability at different time points. **(D)** Comparison of ASCC3 expression levels between male and female patient samples and normal tissues (*p < 0.05; **p < 0.01; ns, not significant). **(E)** Comparison of ASCC3 expression levels between patient samples ≤65 years and >65 years, as well as between normal tissues (**p < 0.01; ns, not significant). **(F–I)** Comparison of ASCC3 expression levels between different tumor tissues and normal tissues across T stage, N stage, M stage and pathological stage (*p < 0.05; ns, not significant).

We first performed ROC curve analysis using sample data from the TCGA dataset. The results showed an AUC of 0.800 (95% CI: 0.708–0.888). Subsequently, we expanded the sample size by incorporating data from the GTEx database for further diagnostic performance assessment, which yielded an AUC of 0.917 (95% CI: 0.876–0.950). These results suggest that ASCC3 expression exhibits strong diagnostic potential for rectal cancer (Figure 4A; Supplementary Figure S3). Additionally, we conducted time-dependent ROC curve analysis, which showed AUC values of 0.522, 0.319, and 0.229 for 1-year, 3-year, and 5-year overall survival (OS), respectively. Since ASCC3 has been identified as a protective factor for rectal cancer in this study, a smaller area under the curve (AUC), closer to 0, indicates better prognostic diagnostic efficacy (Figure 4B). This suggests that the predictive value of ASCC3 in rectal cancer gradually increases over time. Additionally, to provide a more intuitive display and optimization of prognostic prediction, we plotted the calibration curves of ASCC3 with the 1-year, 3-year, and 5-year overall survival (OS) rates of patients (Supplementary Figure S4). The results showed that, as time progressed, the closer the curve was to the diagonal line, the more it indicated the increasing predictive value of ASCC3 in rectal cancer over time. Finally, we constructed a nomogram combining ASCC3 expression with other clinical features (Figure 4C). Although ASCC3 does not carry as much weight as the N stage and pathological stage, it still holds certain diagnostic value in the overall predictive model.

Additionally, we explored the correlation between ASCC3 expression and clinical features of rectal cancer. As shown in Figures 4D–I, except for age, the expression of ASCC3 in tumor tissues was higher than in normal tissues for gender, T stage, N stage, M stage and pathological stage. However, comparisons within clinical subgroups showed no significant differences. Regarding the age factor, ASCC3 expression within the group showed no significant difference. ASCC3 expression in rectal cancer tissue was higher than in normal tissue for patients <65 years old, while for patients >65 years old, there was no difference in ASCC3 expression between tumor and normal tissues. Therefore, this further supports the reliability of our results where we excluded age as a factor and re-conducted univariate and multivariate Cox regression analysis.

### 3.5 Immune regulatory mechanisms of ASCC3 in rectal cancer

**FIGURE 5:**
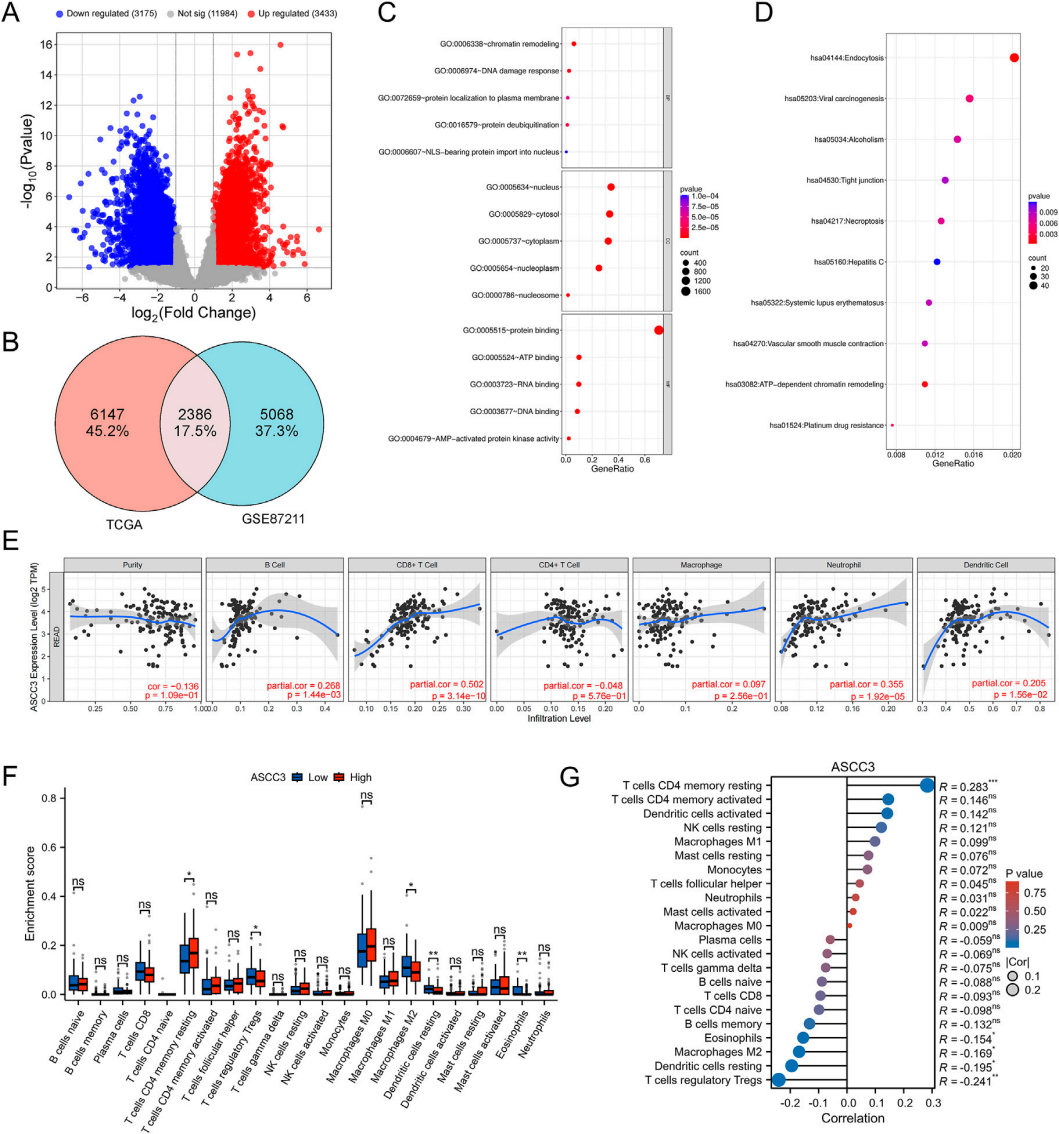
Regulatory mechanisms of ASCC3 in rectal cancer and its correlation with immune cells. **(A)** Volcano plot of differentially expressed genes in TCGA rectal cancer samples. **(B)** Venn diagram illustrating the overlap of differentially expressed genes between TCGA rectal cancer samples and the GSE87211 dataset. **(C)** GO enrichment analysis of common differentially expressed genes from both datasets. **(D)** KEGG enrichment analysis of common differentially expressed genes from both datasets. **(E)** Correlation between ASCC3 expression and six major immune cell types in rectal cancer, as identified using the TIMER database. **(F,G)** CIBERSORT analysis showing the infiltration levels and correlation of ASCC3 expression with 22 immune cell types (*p < 0.05; **p < 0.01; ***p < 0.001; ns, not significant).

To identify genes potentially associated with ASCC3 in rectal cancer and investigate its role in the tumor microenvironment, we selected ASCC3-related differentially expressed genes (DEGs) from TCGA rectal cancer samples. To enhance the reliability and confidence of the results and reduce the degrees of freedom, we also retrieved DEGs from the GSE87211 rectal cancer dataset. The selection criteria were |log2 (FC)|> 1 and p-value <0.05 (Figure 5A). We then performed an intersection of the DEGs from both datasets and conducted GO and KEGG enrichment analyses, with the results arranged in ascending order of p-value (Figures 5B–D). GO enrichment results show that ASCC3-related DEGs are primarily associated with processes such as chromatin remodeling and DNA damage repair in biological processes (BP), are predominantly located in the nucleus and cytoplasm in cellular components (CC), and are involved in processes like protein, ATP, DNA and RNA binding in molecular functions (MF). KEGG enrichment results reveal that DEGs are mainly involved in processes such as cell endocytosis, viral carcinogenesis, alcohol intoxication, epithelial cell tight junctions and necroptosis.

To further investigate the mechanism of ASCC3 in the tumor immune microenvironment of rectal cancer, we first analyzed the relationship between ASCC3 and six common immune cell types in rectal cancer using the TIMER database. The results showed a significant positive correlation between ASCC3 and CD8^+^ T cells, while correlations with other immune cells were weaker (Figure 5E). We then further evaluated ASCC3 infiltration in 22 immune cell types using CIBERSORT analysis. Since CIBERSORT uses the LM22 normalized matrix, which includes gene expression profiles for 22 immune cell types based on 547 genes, it allows for a more refined analysis of ASCC3’s association with immune cells. The results indicated that the high-expression group of ASCC3 showed higher infiltration of resting memory CD4^+^ T cells, while the low-expression group exhibited more significant infiltration of M2 macrophages and regulatory T cells (Tregs). Correlation analysis showed a significant positive correlation between ASCC3 and resting memory CD4^+^ T cells, while negative correlations were observed with Tregs, M2 macrophages, and other immune cells (Figures 5F,G). However, no correlation was observed with CD8^+^ T cells. Therefore, we used the EMTAB8107 single-cell dataset to validate the relationship between ASCC3 and CD8^+^ T cells (Supplementary Figure S5). The results showed that ASCC3 was significantly highly expressed in malignant cells, exhausted CD8^+^ T cells, and conventional CD4^+^ T cells, which also validated some of the findings from the TIMER and CIBERSORT analyses. Therefore, we hypothesize that ASCC3 exerts its antitumor immune effects and provides protective effects primarily by coordinating the immune roles of different types of CD4^+^ T cells, CD8^+^ T cells, M2 macrophages, and Tregs in the tumor microenvironment.

### 3.6 ASCC3 can exert a protective effect in conjunction with various immune-related genes

**FIGURE 6:**
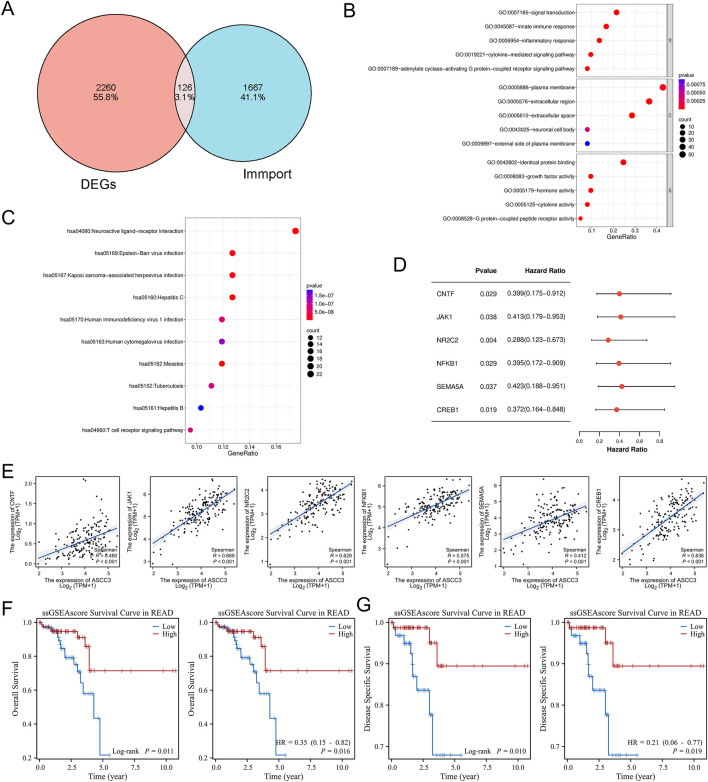
ASCC3 influences patient prognosis in conjunction with various immune-related genes. **(A)** Venn diagram showing the intersection of differentially expressed genes (DEGs) from both datasets and immune-related genes (IRGs) from the IMMPORT database. **(B,C)** GO and KEGG enrichment pathways of immune-related genes among the common DEGs. **(D)** Immune-related genes associated with ASCC3 that affect the prognosis of rectal cancer patients. **(E)** Correlation analysis between ASCC3 and immune-related genes affecting the prognosis of rectal cancer patients, including CNTF, JAK1, NR2C2, NFKB1, SEMA5A and CREB1. **(F,G)** Analysis of Overall Survival (OS) and Disease-Specific Survival (DSS) of rectal cancer patients using an independent gene set formed by ASCC3, CNTF, JAK1, NR2C2, NFKB1, SEMA5A and CREB1.

To further investigate the immune regulatory mechanisms involved by DEGs, we intersected the DEGs with immune-related genes (IRGs) obtained from the IMMPORT database (Figure 6A), and performed GO and KEGG enrichment analyses on the 126 common genes, presenting the results in order of increasing p-value (Figures 6B,C). GO enrichment analysis revealed that these common genes were primarily associated with signal transduction, innate immune response, and inflammation response in biological processes (BP), located mainly in the plasma membrane and extracellular regions in cellular components (CC), and involved in protein binding, growth factor activity, hormone activity and cytokine activity in molecular functions (MF). KEGG enrichment analysis showed that these common genes were involved in the T cell receptor signaling pathway, which also corroborated the previous results. Therefore, we infer that ASCC3 mainly enhances immune function by modulating T cell immune activity, thereby exerting a protective anti-tumor effect.

Additionally, we performed Cox regression analysis to assess the impact of the 126 immune-related genes on the prognosis of rectal cancer patients, as shown in Figure 6D. Six immune-related genes, including JAK1, NFKB1, SEMA5A, NR2C2, CNTF and CREB1, passed the Cox regression test with p-value <0.05 and hazard ratios (HR) < 1. This further validates ASCC3 as a favorable factor in rectal cancer. We hypothesize that ASCC3 exerts its protective effect on rectal cancer patients by regulating the functions of these six immune-related genes. Furthermore, Spearman correlation analysis of ASCC3 and these six immune-related genes showed significant positive correlations between them (Figure 6E). To further confirm these findings, we created an independent gene set sample by combining ASCC3 and these six immune-related genes and constructed an ssGSEA pathway score. We then tested their association with the overall survival (OS) and disease-specific survival (DSS) of rectal cancer patients. The results indicated that higher pathway scores associated with ASCC3 were linked to better OS and DSS. Additionally, higher pathway scores were also a favorable prognostic factor (Figures 6F,G). Thus, these results further demonstrate that ASCC3 can exert a protective effect on rectal cancer patients by cooperating with multiple immune-related genes.

### 3.7 Prognostic model for ASCC3 and multiple genes using machine learning algorithms

**FIGURE 7:**
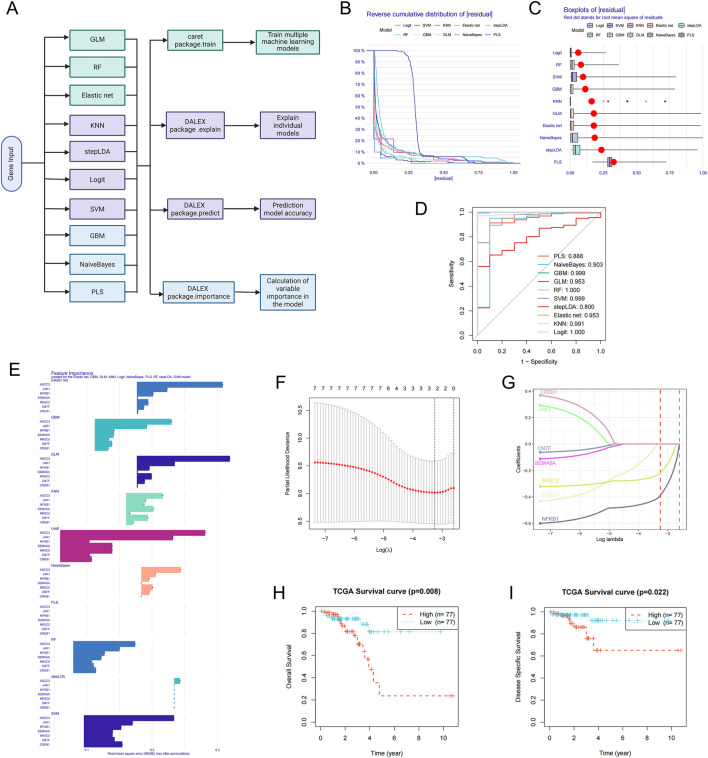
Construction of prognostic models using multiple machine learning algorithms. **(A)** Architecture diagram of machine learning for prognostic diagnosis. **(B)** Residual reverse cumulative distribution plot of samples. The x-axis represents the residual values, ranging from the minimum to the maximum. The y-axis represents the cumulative frequency of residuals, ranging from 1 (all samples) to 0 (no samples). **(C)** The residual boxplot displays the median, interquartile range (IQR, the difference between the third and first quartiles), and outliers of the residuals. **(D)** ROC curve evaluating the performance of the binary classifier. **(E)** Variable importance ranking analysis plot, showing the genes ranked by importance for each model. **(F)** Ten-fold cross-validation result plot, displaying cross-validation errors under different λ values. **(G)** Lasso regression coefficient path plot. **(H,I)** Kaplan-Meier survival analysis plots for Overall Survival (OS) and Disease-Specific Survival (DSS), with red representing the high-risk group and blue representing the low-risk group.

To further evaluate the prognostic value of ASCC3 in combination with six immune-related genes (JAK1, NFKB1, SEMA5A, NR2C2, CNTF and CREB1) for rectal cancer patients, we constructed diagnostic and prognostic models using various machine learning algorithms (Figure 7A), including Generalized Linear Model (GLM), Random Forest (RF), Elastic Net, K-Nearest Neighbors (KNN), Stepwise Linear Discriminant Analysis (stepLDA), Logistic Regression (Logit), Support Vector Machine (SVM), Gradient Boosting Machine (GBM), Naive Bayes (NaiveBayes), and Partial Least Squares (PLS). As shown in Figures 7B–D, the residual cumulative distribution, residual boxplot, and ROC curve results all indicate the model’s strong diagnostic performance. Variable importance analysis highlights ASCC3 as the most significant predictor in the model (Figure 7E). As illustrated in Figures 7F,G, ASCC3 approaches the optimal λ value more closely than the other genes, further confirming its pivotal role in the prediction model. Moreover, Kaplan-Meier survival analysis of Overall Survival (OS) and Disease-Specific Survival (DSS) in high- and low-risk groups demonstrates the clinical relevance of this prognostic model (Figures 7H,I).

### 3.8 ASCC3 is a specific protective factor in rectal cancer among various types of digestive system cancers

**FIGURE 8:**
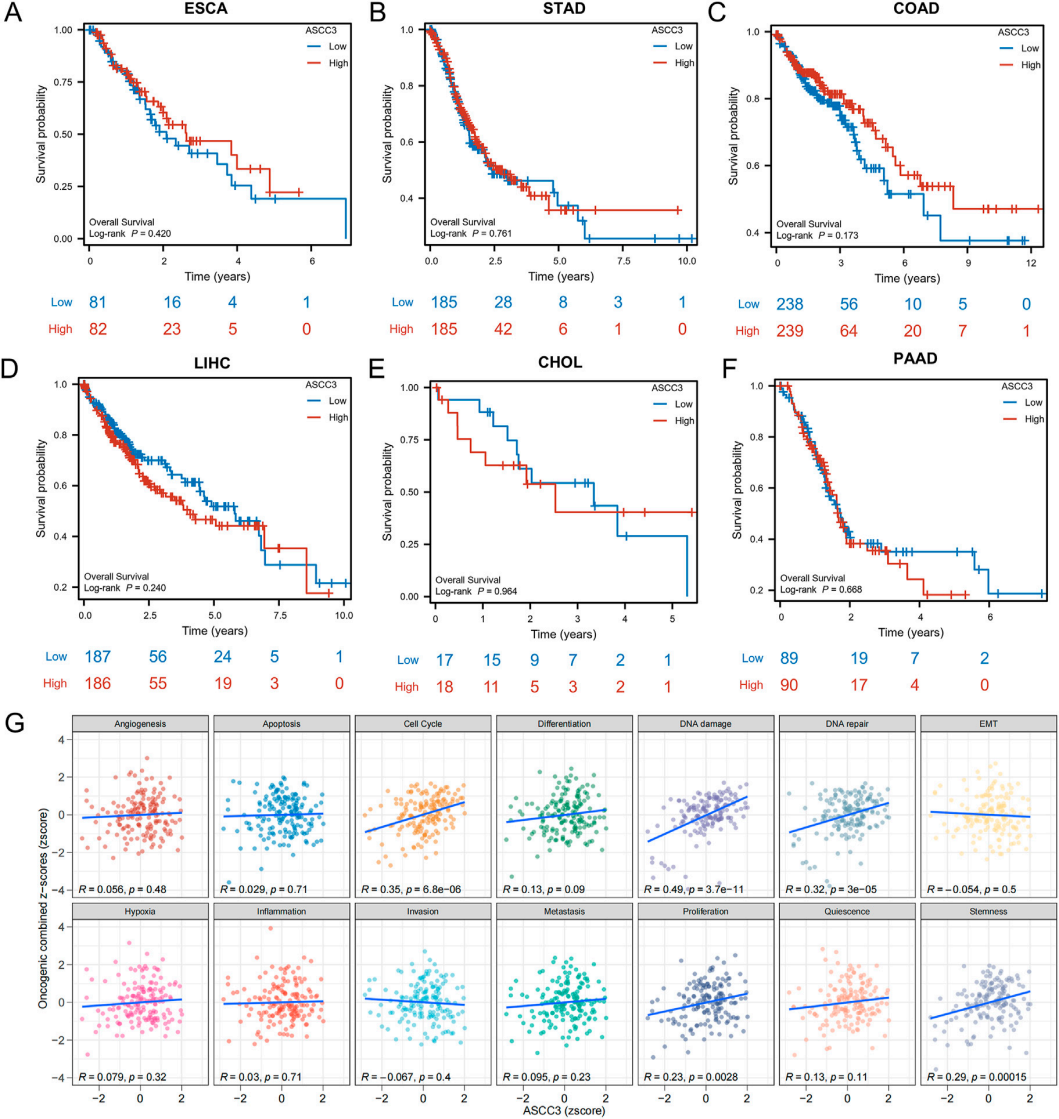
Association of ASCC3 expression with Overall Survival (OS) in various digestive system cancers and Gene Set Variation Analysis (GSVA) analysis. **(A–F)** Correlation between ASCC3 expression and survival in different digestive system cancers, including esophageal carcinoma (ESCA), stomach adenocarcinoma (STAD), colon adenocarcinoma (COAD), liver hepatocellular carcinoma (LIHC), cholangiocarcinoma (CHOL) and pancreatic adenocarcinoma (PAAD). **(G)** GSVA evaluates the correlation between ASCC3 expression and the functional status scores of 14 cancer cell types.

As shown in Figures 8A–F, we analyzed the impact of ASCC3 expression on the prognosis of patients with various digestive system cancers. The results indicated that ASCC3 expression had no impact on the OS of patients with esophageal carcinoma (ESCA), stomach adenocarcinoma (STAD), colon adenocarcinoma (COAD), liver hepatocellular carcinoma (LIHC), cholangiocarcinoma (CHOL) and pancreatic adenocarcinoma (PAAD). We also analyzed the association between ASCC3 expression and prognosis in other cancer types. The results showed that high ASCC3 expression was a protective factor for patients with kidney renal clear cell carcinoma (KIRC), while it was a risk factor for patients with cervical squamous cell carcinoma and endocervical adenocarcinoma (CESC) (Supplementary Figure S6). In addition, we also evaluated the association between ASCC3 expression and patient OS in various digestive system cancers (Supplementary Table S1). Based on the aforementioned multiple validations of ASCC3 in rectal cancer prognosis, we conclude that ASCC3 is a specific protective factor in rectal cancer among various digestive system cancers.

### 3.9 The specific role of ASCC3 in rectal cancer

To investigate the role of ASCC3 in various types of digestive system cancers, we performed GSVA analysis to examine the correlation between ASCC3 expression and different functional states in 14 types of cancer. The results indicated a significant positive correlation between ASCC3 expression and scores for the cell cycle, DNA damage and DNA repair functional states (Figure 8G). We also assessed the association of ASCC3 expression with these functional states in multiple digestive system cancers, and the results showed that ASCC3 exhibited a stronger correlation with these states in rectal cancer compared to other digestive system cancers (Supplementary Figure S7). Therefore, we hypothesize that ASCC3 may exert a specific protective effect in rectal cancer by modulating stronger cell cycle regulation, DNA damage and DNA repair.

### 3.10 Mutation analysis of ASCC3 across pan-cancer

**FIGURE 9:**
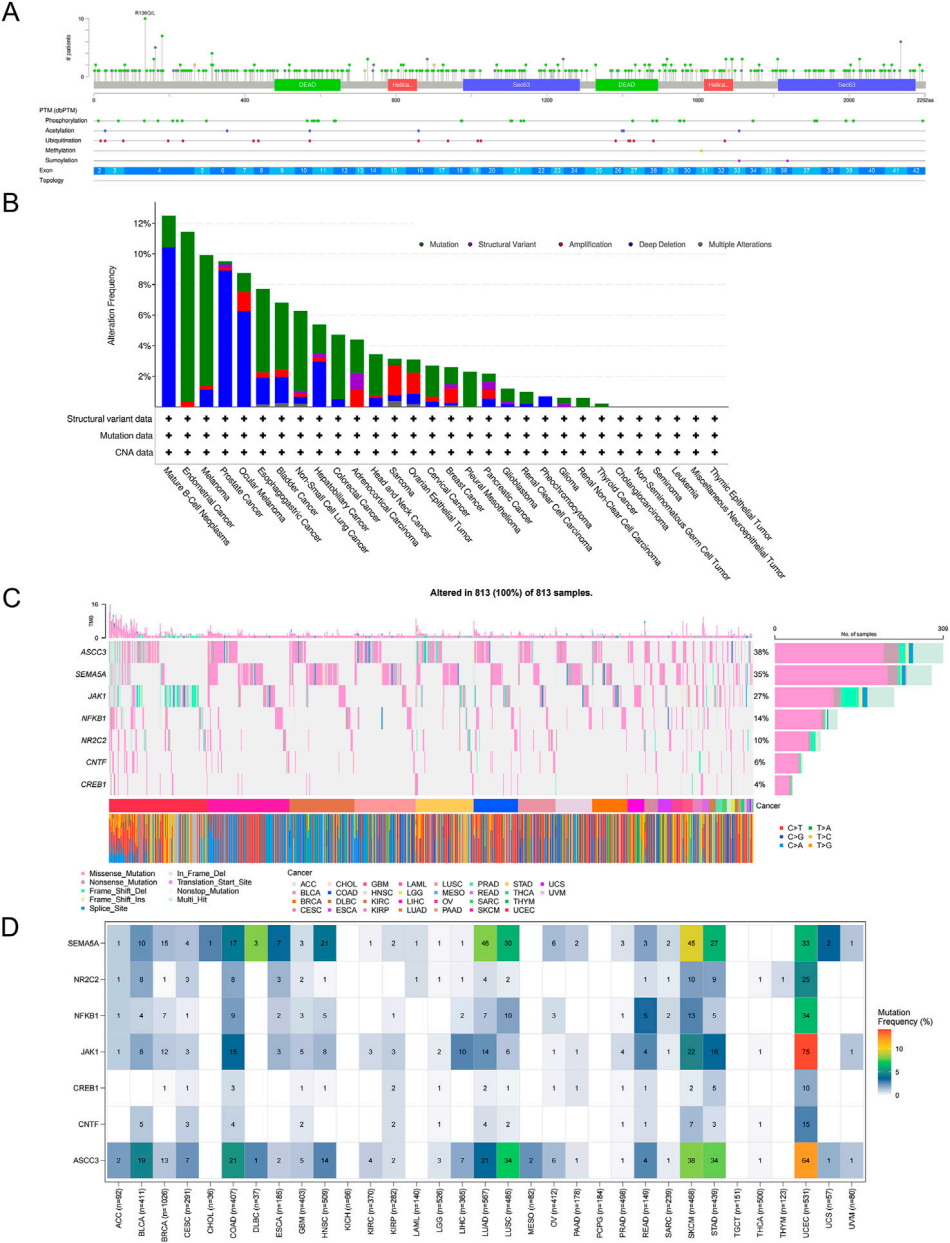
Mutation analysis of ASCC3 in pan-cancer. **(A)** Bar plot of ASCC3 gene mutation sites in pan-cancer. **(B)** Mutation frequency plot of different mutation types of ASCC3 in pan-cancer. **(C,D)** Mutation waterfall plot and mutation frequency plot of ASCC3, SEMA5A, JAK1, CNTF, NFKB1, NR2C2 and CREB1 genes in pan-cancer.

To investigate the mutation distribution of the ASCC3 gene across pan-cancer, we first explored the mutation hotspots of ASCC3 using the PFAM database (Figure 9A), and subsequently analyzed the mutation frequency differences of ASCC3 across pan-cancer based on the Cbioportal database (Figure 9B). Furthermore, we analyzed the mutation status of ASCC3, JAK1, NFKB1, SEMA5A, NR2C2, CNTF and CREB1 across pan-cancer. The results showed that ASCC3, SEMA5A, and JAK1 exhibited higher mutation frequencies across pan-cancer, whereas among specific cancer types, these seven genes had relatively lower mutation frequencies in rectal cancer, but a higher mutation frequency in endometrial cancer (Figures 9C,D).

### 3.11 Signaling pathways and networks involving ASCC3

**FIGURE 10:**
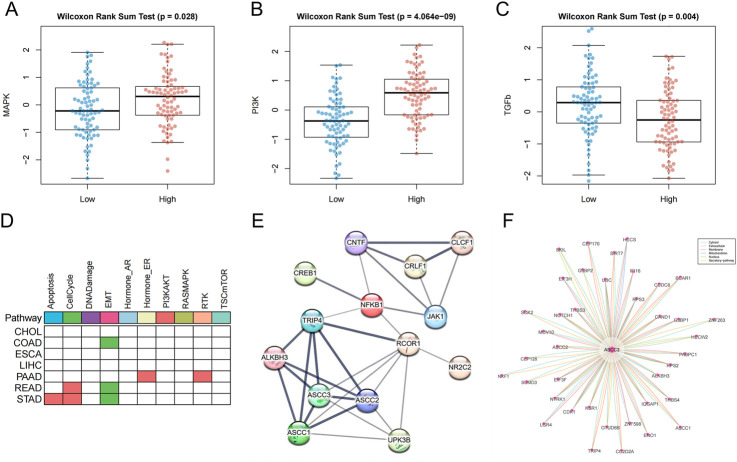
Pathway analysis and interaction network analysis related to ASCC3. **(A)** ASCC3 is highly expressed in the MAPK signaling pathway. **(B)** ASCC3 is highly expressed in the PI3K signaling pathway. **(C)** ASCC3 is lowly expressed in the TGF-β signaling pathway. **(D)** The differential activity of cancer pathways with high ASCC3 expression in different digestive system cancers. Red indicates higher pathway activity in the high-expression group, green represents lower pathway activity in the high-expression group, and white indicates no significance. **(E)** ASCC3 interaction network analysis based on the STRING database (line thickness indicates the strength of data support). **(F)** ASCC3 interaction network analysis based on the ComPPI database.

To further explore the mechanisms and molecular interactions involved in ASCC3 within the tumor microenvironment, we analyzed the differences in ASCC3 expression across 14 tumor-associated signaling pathways, including androgen, EGFR, estrogen, hypoxia, JAK-STAT, MAPK, NF-κB, p53, PI3K, TGF-β, TNF-α, TRAIL, VEGF and WNT signaling pathways. The results indicated that high ASCC3 expression was associated with the activation of the MAPK and PI3K pathways, while low ASCC3 expression was linked to the activation of the TGF-β pathway (Figures 10A–C; Supplementary Figure S8).

In addition, we downloaded the protein expression data for ASCC3 from the TCPA database and calculated the differences in the activity scores of 10 cancer pathways associated with high ASCC3 expression. The results showed that the cell cycle pathway was activated in both gastric and rectal cancers, while the EMT pathway remained suppressed in gastric, colon, and rectal cancers. Given that the MAPK and PI3K pathways are primarily involved in cell growth, proliferation, and metabolism, while the cell cycle pathway regulates cell proliferation to ensure accurate replication and distribution of genetic material to offspring, maintaining genomic stability. Additionally, the TGF-β pathway can coordinate with the EMT pathway to promote cancer cell migration and invasion. This finding indirectly suggests how high ASCC3 expression may contribute to a protective role in rectal cancer (Figure 10D; Supplementary Figure S9). Furthermore, we utilized the STRING and ComPPI databases to explore the associations between ASCC3 and ASCC3, JAK1, NFKB1, SEMA5A, NR2C2, CNTF and CREB1 genes, as well as other proteins that may interact with ASCC3 (Figures 10E,F). Finally, we used the BioGRID database to identify proteins with ASCC3 as both a source and target node (Supplementary Figure S10).

## 4 Discussion

Increasing evidence suggests that ASCC3 is involved not only in DNA damage repair but also in the immune regulation of tumors (Ao et al., 2023; Li et al., 2013). Our study reveals that ASCC3 expression is elevated in various cancers, including rectal adenocarcinoma, compared to normal tissues, suggesting its amplificatory role in cancer. Additionally, we validated these findings using the GEO database. Furthermore, ASCC3 expression shows significant prognostic differences across various cancer types. Our findings indicate that rectal cancer patients with high ASCC3 expression tend to have a better prognosis compared to those with other digestive system cancers. Subgroup analysis of the rectal cancer cohort suggests that high ASCC3 expression is associated with better prognosis, particularly in late-stage, non-metastatic patients, especially among females. Additionally, our diagnostic and prognostic models, constructed using machine learning, highlight the significant predictive value of ASCC3 in combination with various immune-related genes, including JAK1, NFKB1, SEMA5A, NR2C2, CNTF and CREB1, for rectal cancer prognosis.

However, the role of ASCC3 in cancer remains unclear. Its specific function likely varies depending on cancer type, molecular mechanisms, and the tumor microenvironment (Jia J. et al., 2020; Jia M. et al., 2020). Under normal conditions, ASCC3 is involved in DNA damage repair and RNA metabolism, such as pre-mRNA splicing, to maintain genomic stability. However, in certain cases, the loss of ASCC3 function may lead to abnormal RNA processing or the accumulation of DNA damage, resulting in genomic instability. Furthermore, aberrant repair activity may foster the accumulation of mutations or resistance to radiotherapy and chemotherapy. Relevant studies suggest that high ASCC3 expression may repair chemotherapy-induced DNA damage, enhancing cancer cell survival and indirectly promoting tumor progression (Cao et al., 2025). Our study demonstrates that ASCC3 can serve as an independent prognostic biomarker for rectal cancer, particularly in younger patient populations. Given that ASCC3 plays a pivotal role in DNA damage repair, the genomic repair capacity and stability in elderly patients significantly decline with age. Additionally, the mutation frequency in aging individuals increases substantially, exceeding the regulatory control of ASCC3. As a result, the prognostic capability of ASCC3 is attenuated in elderly patients compared to their younger counterparts. However, current research indicates that a substantial proportion of early-onset rectal cancer occurs in younger individuals, and the demographic of rectal cancer patients is progressively becoming younger. Over the past 20 years, the incidence of rectal cancer among the elderly has significantly decreased in many countries. However, the incidence of early-onset rectal cancer in those under 65, especially under 50, has doubled (Díaz-Gay et al., 2025; Siegel et al., 2019; Vuik et al., 2019). Therefore, ASCC3 retains considerable diagnostic and prognostic value, with significant implications for future research in rectal cancer.

The role of ASCC3 varies across different cancer types, which may be related to its function in DNA repair and maintaining genomic stability. In our pan-cancer prognostic study, we found that high ASCC3 expression serves as a protective factor for patients with clear cell renal carcinoma, while it acts as a risk factor for cervical cancer patients. We hypothesize that this may be related to the growth and differentiation rates of tissue cells. Since the growth and differentiation of rectal and renal clear cells are relatively slow, high ASCC3 expression can typically repair cellular dysfunction and maintain genomic stability when cells are disturbed by external stimuli or intrinsic regulation. However, when cell growth and differentiation exceed controllable limits, such as in the rapid turnover of cervical epithelial cells, ASCC3’s repair function may become dysregulated, allowing cancer cell proliferation to exploit this mechanism for immune evasion. Our mutational analysis of ASCC3 across various cancers suggests that ASCC3 mutation frequency is relatively low in rectal cancer. Thus, the high genetic stability of ASCC3 may facilitate its role in maintaining DNA damage repair, thereby exerting the protective effect. The positive correlation between ASCC3 and DNA damage and repair in rectal cancer, as well as its activation of the cell cycle and inhibition of the EMT pathway, further supports this notion. Furthermore, our study indicates that high ASCC3 expression is associated with the activation of the MAPK and PI3K pathways, while low ASCC3 expression is linked to the TGF-β pathway. These pathways are known to be involved in cell proliferation and differentiation (Kim and Choi, 2010; Zhang et al., 2017; Zhou et al., 2023), suggesting that ASCC3 may also affect the tumor microenvironment by regulating cell proliferation and differentiation.

Immune regulation is closely associated with tumor progression, with immune cells playing a central role. CD4^+^ T cells, as the commanders of the immune system, assist in the activation of various immune cells and stabilize the function of different immune cell types. They can also differentiate into memory CD4^+^ T cells, which help maintain long-term immune protection. CD8^+^ T cells directly recognize relevant antigens, such as mutated proteins, on the surface of tumor cells to directly kill tumor cells or inhibit tumor growth through IFN-γ (Seder and Ahmed, 2003; St Paul and Ohashi, 2020; Sun et al., 2023). However, immune responses also require some negatively-regulated immune cells to maintain balance. In the tumor microenvironment, these cells can become accomplices in tumor progression and immune evasion. For example, regulatory T cells (Tregs) can suppress the tumor-killing activity of CD8^+^ T cells (Noyes et al., 2022; Zhou et al., 2024), while M2-type tumor-associated macrophages promote tumor immune evasion by secreting immunosuppressive factors (IL-10, TGF-β) and facilitating angiogenesis and matrix remodeling (Shapouri-Moghaddam et al., 2018).

In our analysis of ASCC3 immune infiltration and single-cell sequencing, we found that ASCC3 is associated with immune-activating cells, such as resting CD4^+^ memory T cells and conventional CD4^+^ T cells, in the tumor immune microenvironment. At the same time, it is also linked to immune-suppressive cells, including exhausted CD8^+^ T cells, M2 macrophages, and Tregs. This suggests that ASCC3 may mediate antitumor immune responses by coordinating the functions of various immune cells, thereby exerting a protective effect in rectal adenocarcinoma patients. This was further confirmed by our finding that immune gene sets associated with ASCC3 are involved in the T-cell receptor signaling pathway. This suggests that ASCC3 may participate in repairing damage to immune-positive regulatory cells associated with T cells, thereby maintaining anti-tumor immune efficacy and inhibiting tumor immune evasion (Barnett and Barnett, 1998). Immune-related genes also play a crucial role in maintaining the host’s immune function. Our study shows that multiple immune genes associated with ASCC3, including JAK1, NFKB1, SEMA5A, NR2C2, CNTF and CREB1, positively impact the prognosis of rectal adenocarcinoma patients. Whether these immune-related genes can be used as therapeutic targets for immune treatment in rectal cancer warrants further investigation.

This study demonstrates that high expression of ASCC3 can protect rectal adenocarcinoma patients by maintaining genomic stability through its DNA damage repair function, as well as by participating in the regulation of the cell cycle, proliferation, and differentiation. ASCC3 can serve as a prognostic biomarker for rectal cancer. ASCC3, in conjunction with various immune-related genes, may influence patient prognosis and potentially enhance anti-tumor immunity by regulating T cell immune functions. However, this study also has limitations. First, as it relies on bioinformatics analysis and is retrospective in nature, caution must be exercised when interpreting the results. Furthermore, although this study reveals the protective role of ASCC3 in rectal cancer patients, it does not explore how ASCC3 influences the tumor immune microenvironment by regulating immune-related genes and T cell immune functions. To gain a more comprehensive understanding of the immune regulatory mechanisms of ASCC3 in rectal cancer, future studies should incorporate more basic experimental validation.

## 5 Conclusion

This study elucidates the protective role of high expression of ASCC3 in rectal cancer patients, suggesting its potential as a prognostic biomarker for rectal cancer. ASCC3 may influence patient prognosis in conjunction with multiple immune-related genes and could enhance anti-tumor immunity by regulating T cell immune functions. The combination of ASCC3 with several immune-related genes, including JAK1, NFKB1, SEMA5A, NR2C2, CNTF and CREB1, plays a significant predictive role in the prognosis of rectal cancer patients.

## Data Availability

The datasets presented in this study can be found in online repositories. The names of the repository/repositories and accession number(s) can be found in the article/Supplementary Material.
