# Supplementary material for: Integrative multi-omics analysis and machine learning reveal the unique role of ASCC3 in combination with various immune-related genes in rectal adenocarcinoma
Source: Front Genet. 2025 Aug 13;16:1614946. doi: 10.3389/fgene.2025.1614946 (PMC12380680; doi:10.3389/fgene.2025.1614946)
Supplement: Supplementary file 1 [file Supplementaryfile1.docx]

**Supplementary Figures and Table**

**
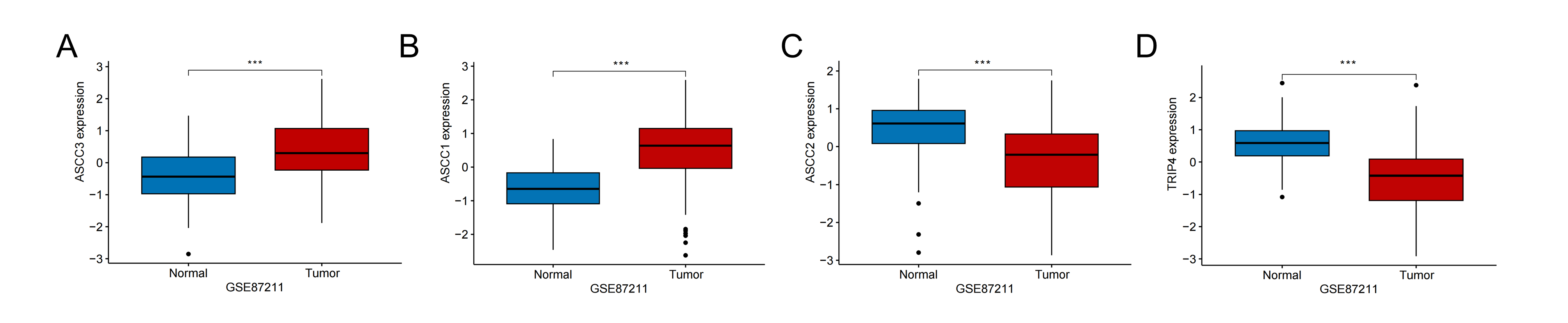
**

**Supplementary Figure S1** Comparison of the expression of ASCC3, ASCC1, ASCC2 and TRIP4 in rectal tumor tissues versus normal tissues in the GSE87211 dataset. **(A)** ASCC3 is highly expressed in tumor tissues (***p < 0.001). **(B)** ASCC1 is highly expressed in tumor tissues (***p < 0.001). **(C)** ASCC2 is highly expressed in normal tissues (***p < 0.001). **(D)** TRIP4 is highly expressed in tumor tissues (***p < 0.001).


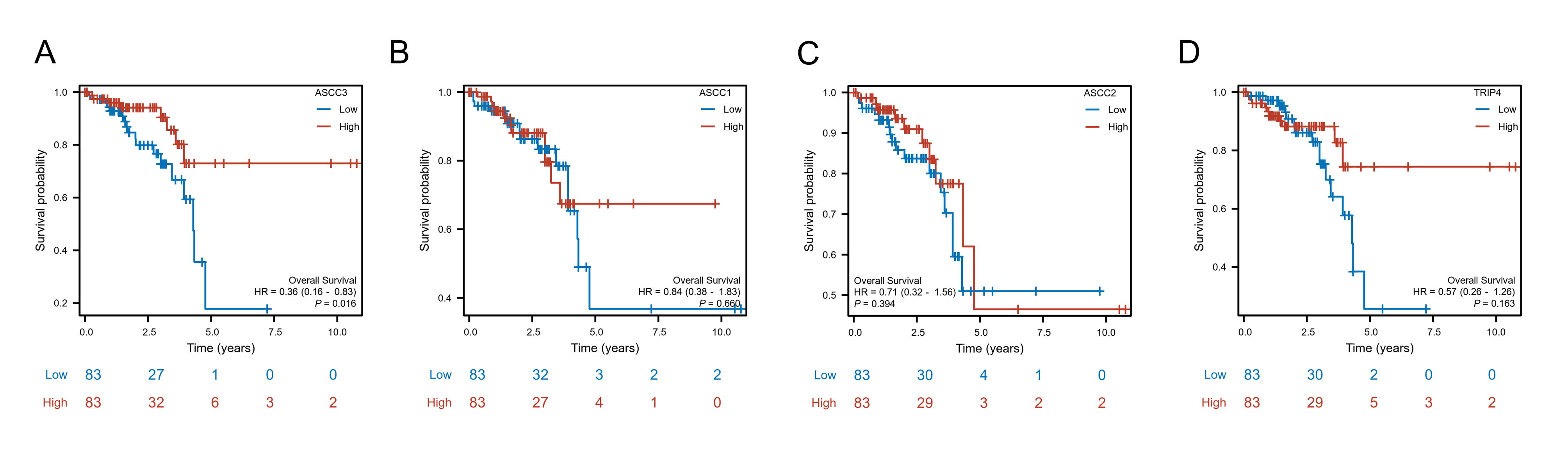


**Supplementary Figure S2** The association between the expression of the four subunits of the ASC-1 complex and overall survival (OS) in rectal cancer patients. **(A)** Patients with high ASCC3 expression in rectal cancer have better survival rates. **(B-D)** The expression of ASCC1, ASCC2 and TRIP4 in rectal cancer is not associated with patient survival.


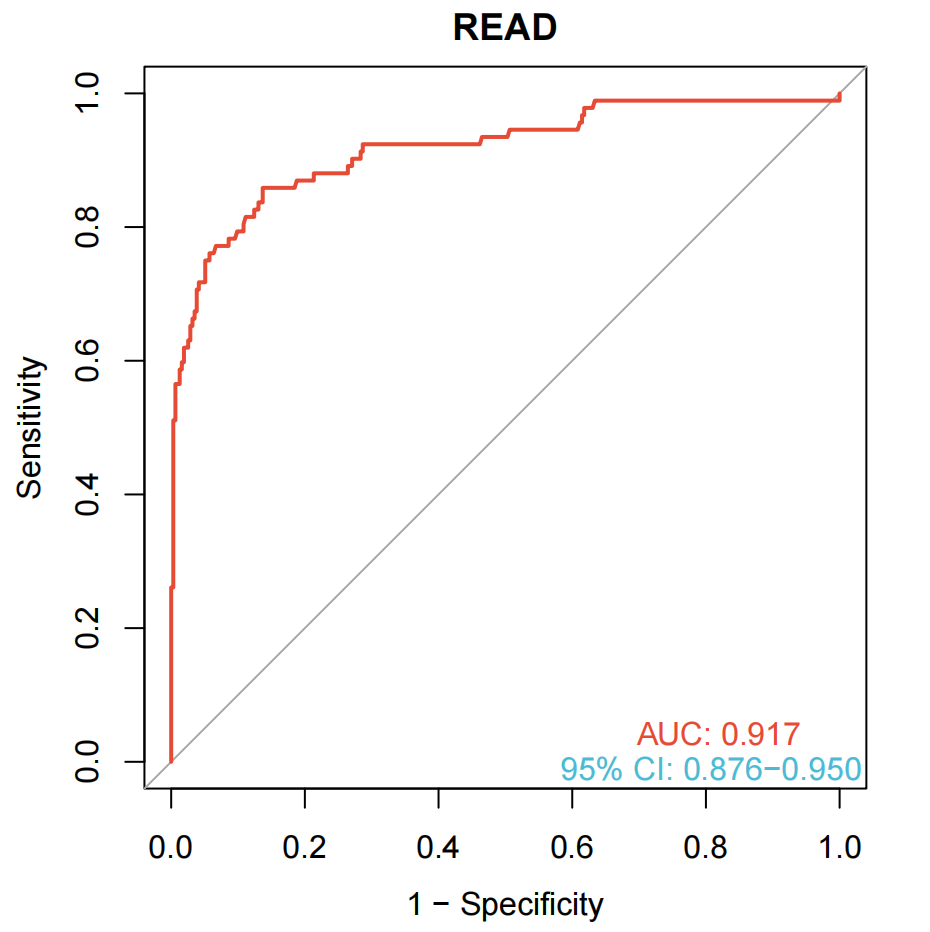


**Supplementary Figure S3** The ROC diagnostic curve was drawn using rectal cancer sample data from TCGA and GTEx. The results show that ASCC exhibits good diagnostic performance (AUC=0.917, 95% CI: 0.876-0.950).


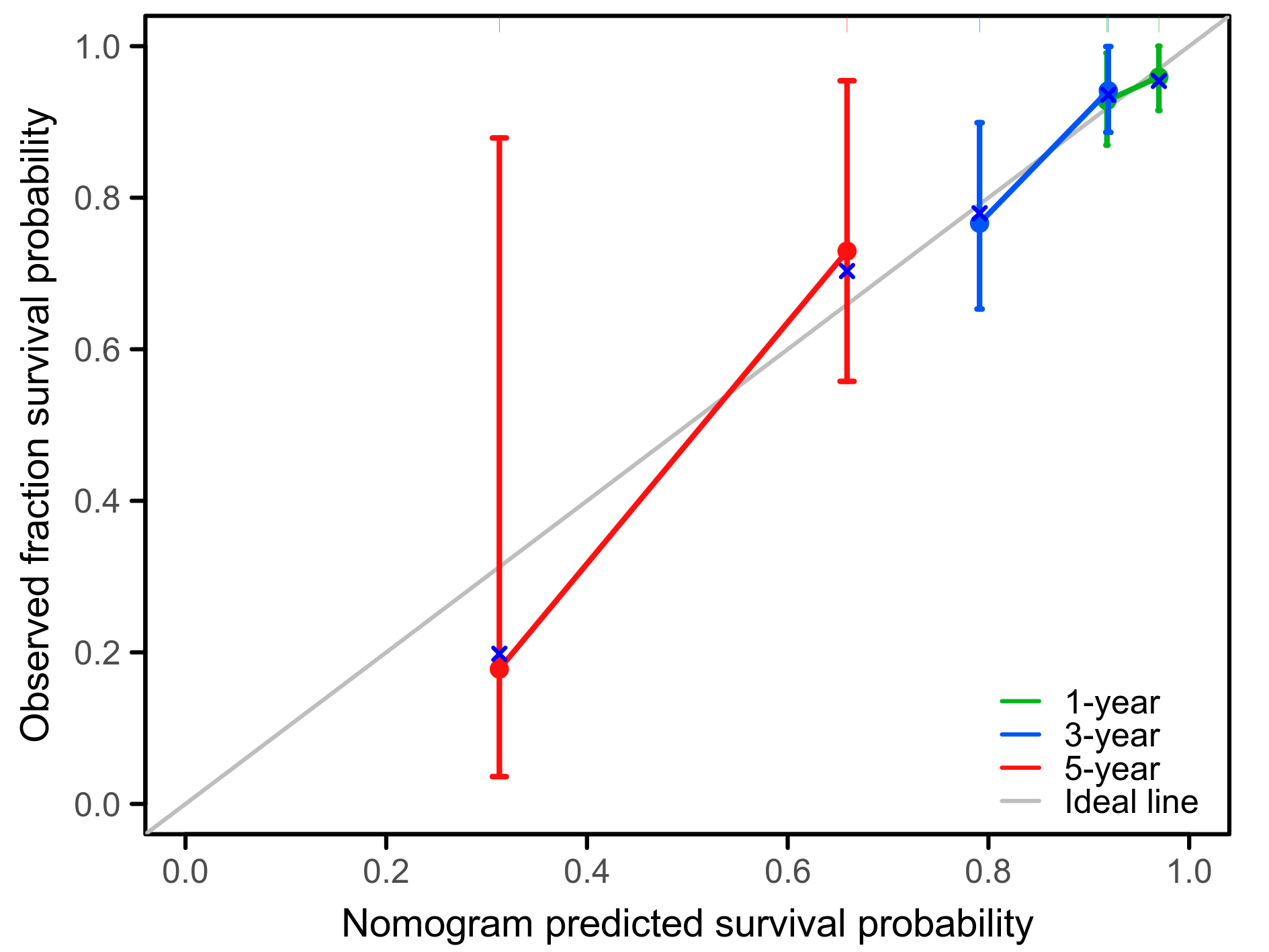


**Supplementary Figure S4** A nomogram predicting the 1-year, 3-year, and 5-year survival probabilities of patients. The horizontal axis represents the survival probabilities predicted by the model, while the vertical axis represents the observed survival probabilities. Each line represents the comparison between predicted and actual survival at specific time points, along with the ideal reference line (the gray diagonal); the closer the line is to the diagonal, the better the model’s calibration.


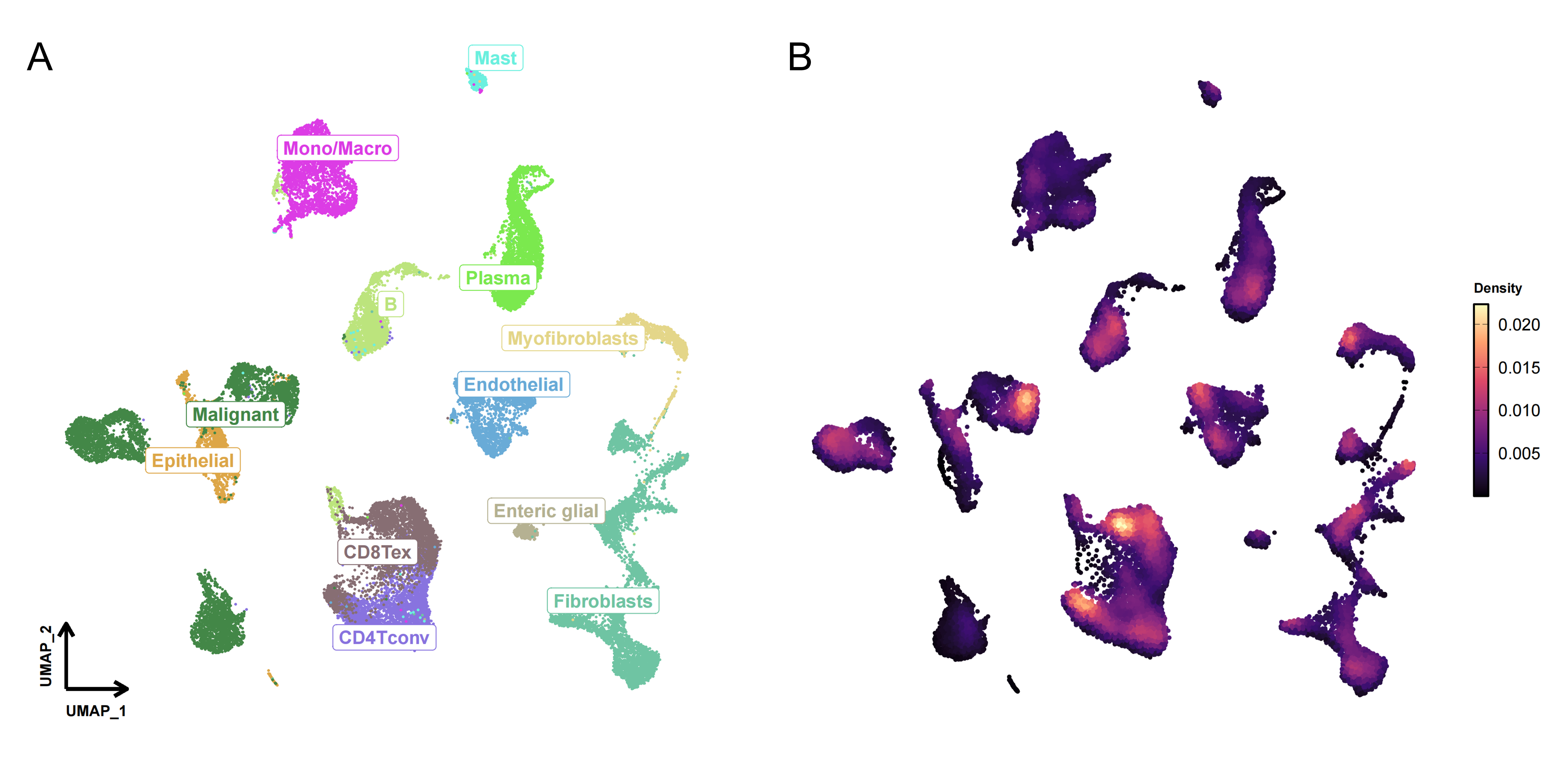


**Supplementary Figure S5** UMAP analysis of the cell and gene data from the EMTAB8107 single-cell dataset indicates that ASCC3 is significantly highly expressed in malignant cells, exhausted CD8^+^ T cells, and conventional CD4^+^ T cells.**（A）**Labeling of different cell types.**（B）**Expression of ASCC3 in different cell types.


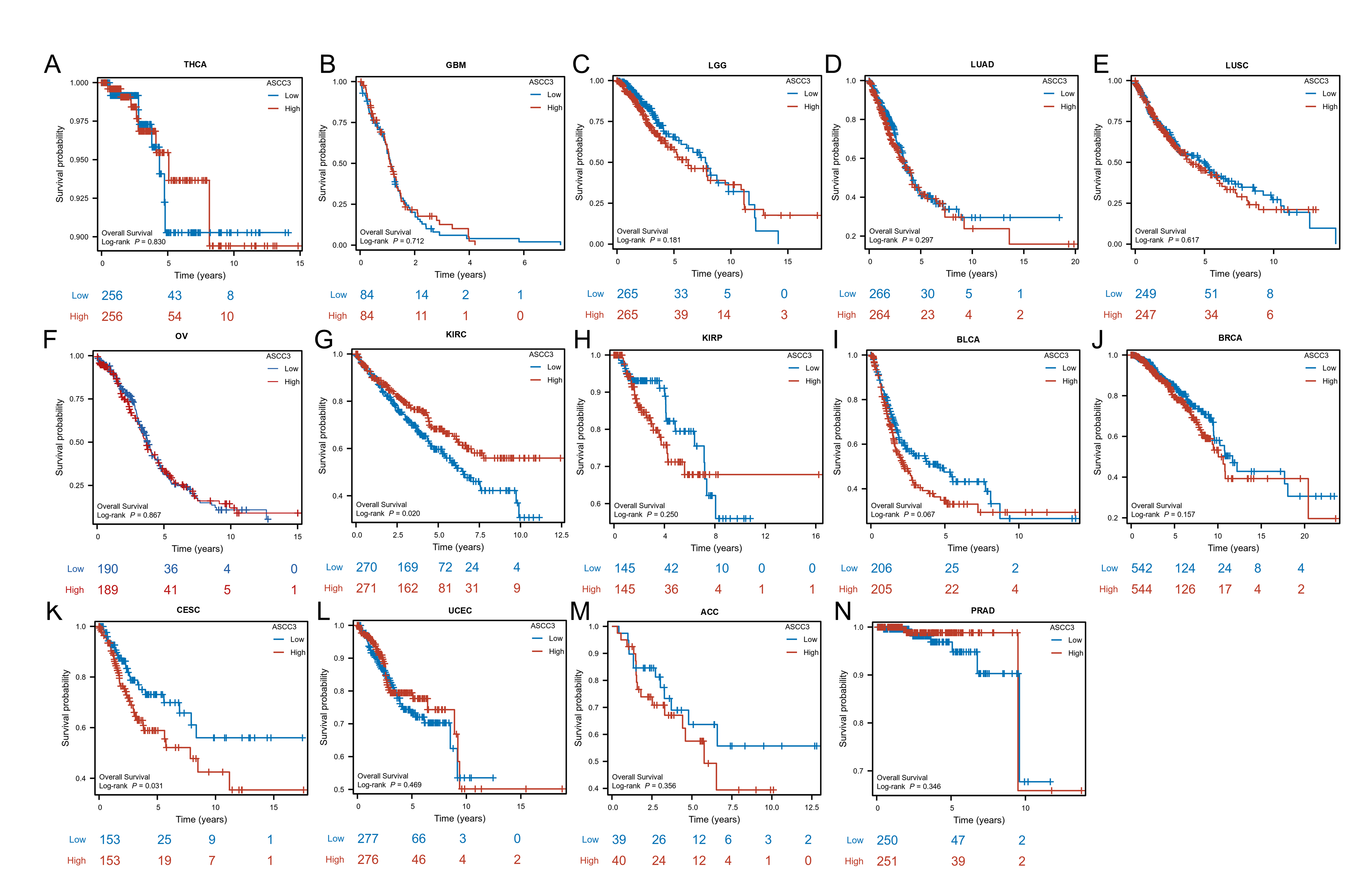


**Supplementary Figure S6** Association of ASCC3 expression with overall survival (OS) in non-digestive system cancers. **(A)** ASCC3 expression is not significantly associated with overall survival in patients with thyroid carcinoma (THCA). **(B)** ASCC3 expression is not significantly associated with overall survival in patients with glioblastoma multiforme (GBM). **(C)** ASCC3 expression is not significantly associated with overall survival in patients with lower grade glioma (LGG). **(D)** ASCC3 expression is not significantly associated with overall survival in patients with lung adenocarcinoma (LUAD). **(E)** ASCC3 expression is not significantly associated with overall survival in patients with lung squamous cell carcinoma (LUSC). **(F)** ASCC3 expression is not significantly associated with overall survival in patients with ovarian serous cystadenocarcinoma (OV). **(G)** Patients with high ASCC3 expression have better survival rates in kidney renal clear cell carcinoma (KIRC). **(H)** ASCC3 expression is not significantly associated with overall survival in patients with kidney renal papillary cell carcinoma (KIRP). **(I)** ASCC3 expression is not significantly associated with overall survival in patients with bladder urothelial carcinoma (BLCA). **(J)** ASCC3 expression is not significantly associated with overall survival in patients with breast invasive carcinoma (BRCA). **(K)** Patients with low ASCC3 expression have better survival rates in cervical squamous cell carcinoma and endocervical adenocarcinoma (CESC). **(L)** ASCC3 expression is not significantly associated with overall survival in patients with uterine corpus endometrial carcinoma (UCEC). **(M)** ASCC3 expression is not significantly associated with overall survival in patients with adrenocortical carcinoma (ACC). **(N)** ASCC3 expression is not significantly associated with overall survival in patients with prostate adenocarcinoma (PRAD).


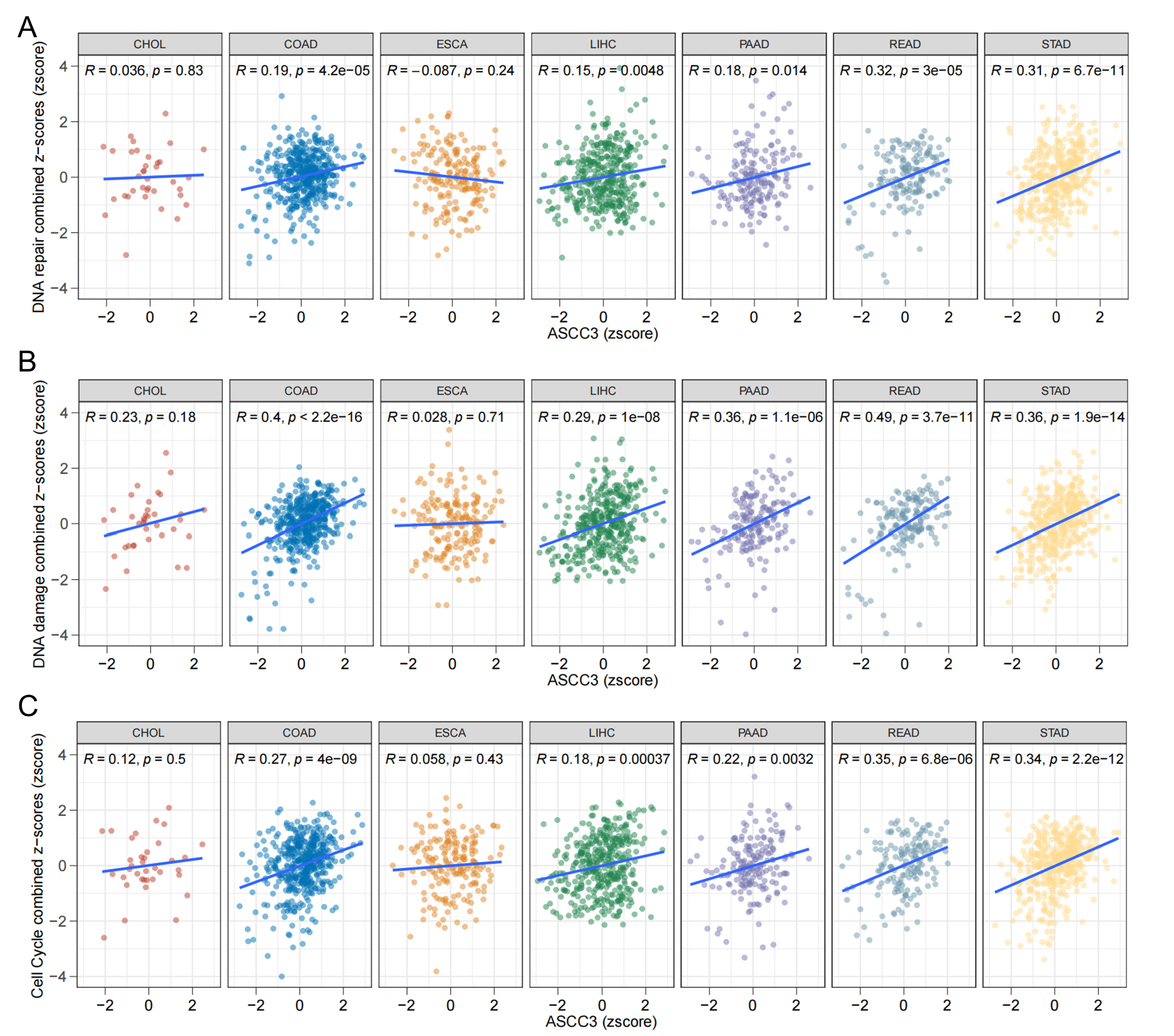


**Supplementary Figure S7** Gene Set Variation Analysis(GSVA) analysis of ASCC3 expression in various cancers. **(A)** GSVA evaluates the correlation between ASCC3 expression and functional status scores of the DNA repair in various digestive system cancers. **(B)** GSVA evaluates the correlation between ASCC3 expression and functional status scores of the DNA damage in various digestive system cancers. **(C)** GSVA evaluates the correlation between ASCC3 expression and functional status scores of the cell cycle in various digestive system cancers.


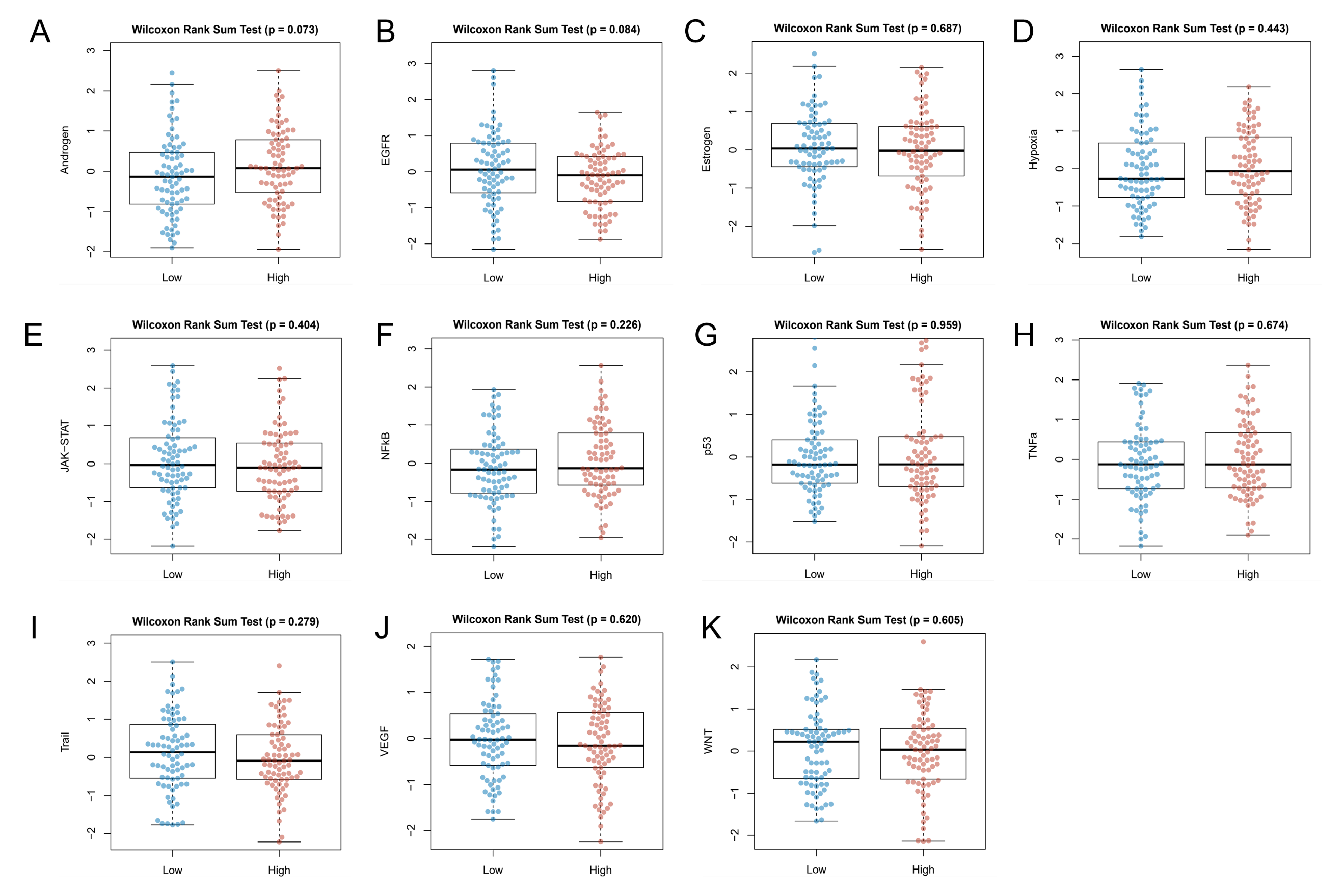


**Supplementary Figure S8** Pathway analysis related to ASCC3. **(A-K)** The correlation between ASCC3 expression levels and the remaining 11 pathways among the 14 tumor-related signaling pathways including androgen, EGFR, estrogen, hypoxia, JAK-STAT, NF-κB, p53, TNF-α, Trail, VEGF and WNT.


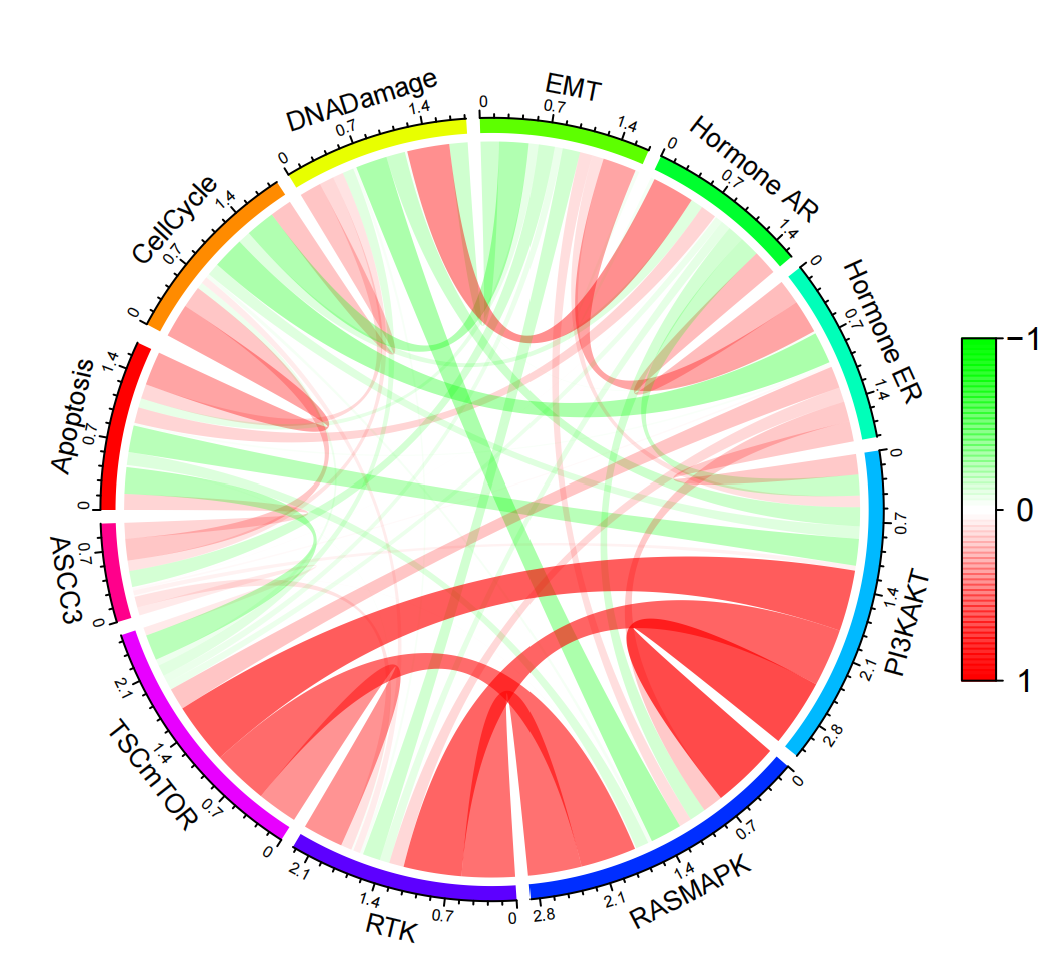


**Supplementary Figure S9** Correlation between ASCC3 expression and pathway-level functional protein quantification from The Cancer Protein Atlas-Revers Phase Protein microArray(TCPA-RPPA) sequencing.


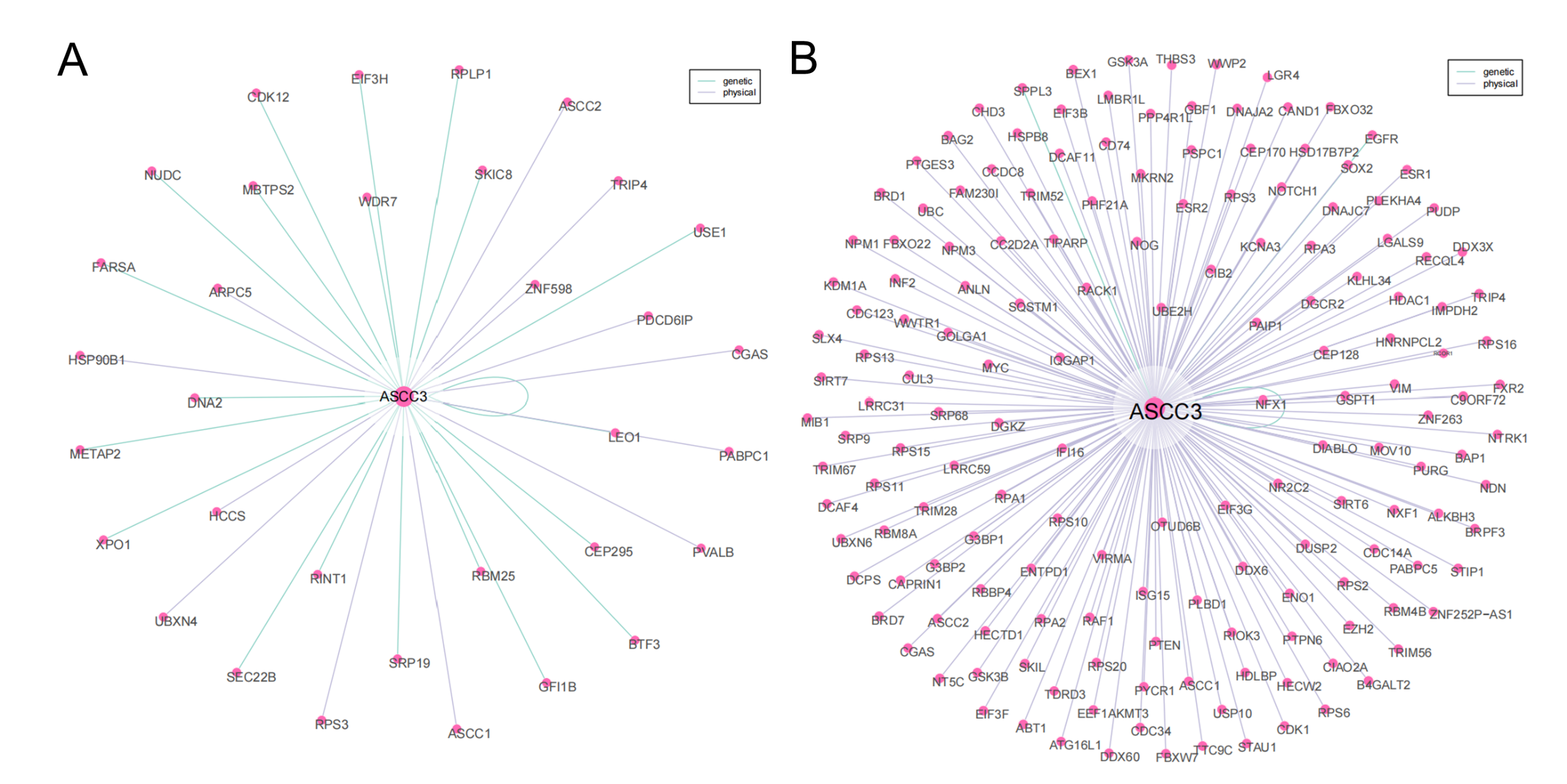


**Supplementary Figure S10** ASCC3 interaction network analysis using ASCC3 as the source protein and target protein based on the BioGRID database. **(A)** Analysis of the interaction network of ASCC3 as a source protein. **(B)** Analysis of the interaction network of ASCC3 as a target protein.

**Supplementary Table S1** Cox regression analysis of survival associated with ASCC3 expression in various types of digestive system cancers.

| Cancer type | Characteristics | Total(N) | HR(95% CI) Univariate analysis | P value Univariate analysis |
| --- | --- | --- | --- | --- |
| ESCA | ASCC3 | 163 | 0.819 (0.503 – 1.333) | 0.421 |
|  | Low | 81 |  |  |
|  | High | 82 |  |  |
| STAD | ASCC3 | 370 | 0.950 (0.685 – 1.320) | 0.762 |
|  | Low | 185 |  |  |
|  | High | 185 |  |  |
| COAD | ASCC3 | 477 | 0.763 (0.517 – 1.127) | 0.174 |
|  | Low | 238 |  |  |
|  | High | 239 |  |  |
| LIHC | ASCC3 | 373 | 1.231 (0.870 – 1.740) | 0.240 |
|  | Low | 187 |  |  |
|  | High | 186 |  |  |
| CHOL | ASCC3 | 35 | 1.022 (0.401 – 2.601) | 0.964 |
|  | Low | 17 |  |  |
|  | High | 18 |  |  |
| PAAD | ASCC3 | 179 | 1.094 (0.725 – 1.651) | 0.669 |
|  | Low | 89 |  |  |
|  | High | 90 |  |  |
